# *Rbfox1* Downregulation and Altered *Calpain 3* Splicing by *FRG1* in a Mouse Model of Facioscapulohumeral Muscular Dystrophy (FSHD)

**DOI:** 10.1371/journal.pgen.1003186

**Published:** 2013-01-03

**Authors:** Mariaelena Pistoni, Lily Shiue, Melissa S. Cline, Sergia Bortolanza, Maria Victoria Neguembor, Alexandros Xynos, Manuel Ares, Davide Gabellini

**Affiliations:** 1 Dulbecco Telethon Institute and Division of Regenerative Medicine, San Raffaele Scientific Institute, Milano, Italy; 2 Department of Molecular, Cell, and Developmental Biology, University of California Santa Cruz, Santa Cruz, California, United States of America; 3 Università Vita-Salute San Raffaele, Milano, Italy; The Jackson Laboratory, United States of America

## Abstract

Facioscapulohumeral muscular dystrophy (FSHD) is a common muscle disease whose molecular pathogenesis remains largely unknown. Over-expression of FSHD region gene 1 (*FRG1*) in mice, frogs, and worms perturbs muscle development and causes FSHD–like phenotypes. *FRG1* has been implicated in splicing, and we asked how splicing might be involved in FSHD by conducting a genome-wide analysis in *FRG1* mice. We find that splicing perturbations parallel the responses of different muscles to *FRG1* over-expression and disease progression. Interestingly, binding sites for the Rbfox family of splicing factors are over-represented in a subset of *FRG1*-affected splicing events. *Rbfox1* knockdown, over-expression, and RNA-IP confirm that these are direct Rbfox1 targets. We find that FRG1 is associated to the *Rbfox1* RNA and decreases its stability. Consistent with this, *Rbfox1* expression is down-regulated in mice and cells over-expressing *FRG1* as well as in FSHD patients. Among the genes affected is *Calpain 3*, which is mutated in limb girdle muscular dystrophy, a disease phenotypically similar to FSHD. In *FRG1* mice and FSHD patients, the *Calpain 3* isoform lacking exon 6 (*Capn3 E6–*) is increased. Finally, *Rbfox1* knockdown and over-expression of *Capn3 E6-* inhibit muscle differentiation. Collectively, our results suggest that a component of FSHD pathogenesis may arise by over-expression of *FRG1*, reducing *Rbfox1* levels and leading to aberrant expression of an altered Calpain 3 protein through dysregulated splicing.

## Author Summary

Alternative splicing is a major contributor to the complexity of human cells, and its disruption can lead to a wide range of human disorders. FSHD is one of the most important muscle diseases. While muscle differentiation defects have been widely reported in the disease, the molecular mechanisms responsible are largely unknown. We found that expression of the alternative splicing factor *Rbfox1* is a direct FRG1 target, and its expression decreased in the muscles of a mouse model of FSHD and FSHD patients. Moreover, alternative splicing of *Calpain 3*, encoding for a protease involved in muscle differentiation, is regulated by Rbfox1 and is altered in the muscles of the mouse model of FSHD and FSHD patients. Interestingly, we found that Rbfox1 is required for muscle differentiation and that this activity is likely mediated by *Calpain 3* alternative splicing. Hence, our results suggest that decreased expression of *Rbfox1* and aberrant *Calpain 3* splicing contribute to the muscle differentiation defects of FSHD patients.

## Introduction

Facioscapulohumeral Muscular Dystrophy (FSHD, OMIM 158900), the third most common myopathy with an incidence of 1 in 15,000 in the human population [1], [2], is characterized by progressive wasting of a specific subset of skeletal muscles [3], [4]. Myogenic defects in FSHD have been widely reported [5]–[11], but the molecular mechanism responsible for them is currently unknown. While FSHD is primarily a disease of skeletal muscles, epilepsy, mental retardation and autism have also been described in severely affected FSHD infants [12]–[15]. FSHD is inherited as an autosomal dominant disorder but is caused by a peculiar molecular mutation [1], [16] involving deletion of tandemly repeated 3.3 kbp sequences, called D4Z4 [17]–[20], in the subtelomeric region of chromosome 4 (4q35). The D4Z4 deletion causes a Polycomb/Trithorax epigenetic switch leading to increased expression of several 4q35 genes specifically in FSHD patients [21]–[23], offering an explanation for its dominant phenotype. Since expression of multiple genes is affected, the molecular pathogenesis of FSHD has been challenging to untangle, and as yet no therapy is available for FSHD patients.

Among the genes up-regulated in FSHD, *FRG1* (FSHD region gene 1) is a likely contributor to FSHD pathogenesis since it is required for normal muscle development [24] and its over-expression in mice, *Xenopus* and *C. elegans* causes an FSHD-like phenotype [24]–[27]. The precise function of FRG1 is still unknown, but there is evidence for a role in RNA processing [25], [28]–[34]. For example, several studies reported association of FRG1 with the spliceosome [28], [30], [32], [33]. Moreover, FRG1 assumes a speckled nuclear distribution pattern characteristic of mammalian splicing factors [34]. Finally, altered splicing of the muscle-expressed genes *MTMR1* and *TNNT3* has been reported in FSHD [25].

Muscle tissues, like brain, are rich in their use of tissue-specific alternative splicing events to regulate gene expression and produce specialized protein isoforms. Many of these events display enrichment for putative binding sites for the evolutionary conserved, tissue-specific Rbfox family of alternative splicing regulators: Rbfox1 (Fox-1 or A2BP1), Rbfox2 (Fox-2 or Rbm9) and Rbfox3 (Fox-3 or NeuN) [35]. *Rbfox1* is expressed in brain, skeletal muscle and heart [35]–[38], while *Rbfox2* has a broader expression pattern, being detected in whole embryo, stem cells, hematopoietic cells and in adult brain, heart and ovary [35], [36], [39]–[42]. In contrast, *Rbfox3* has been observed only in neurons [36], [43]. So far, few genes have been experimentally validated as Rbfox family targets in muscle. In this paper, we show that *FRG1* over-expression in mouse muscle is associated with widespread alternative splicing perturbations that appear to delay or inhibit proper muscle development at a cellular level. We show that FRG1 over-expression decrease *Rbfox1* RNA. In *FRG1* mouse muscles, C2C12 myoblasts over-expressing *FRG1*, and in FSHD patients, down-regulation of *Rbfox1* expression leads to altered splicing of Rbfox1-dependent muscle exons. We further show that Rbfox1 is required for myogenesis and part of this requirement may involve correct regulation of *Calpain 3* alternative splicing. Our results provide a molecular mechanism for myogenic defects in FSHD and identify possible therapeutic targets.

## Results

### Widespread mRNA expression level and alternative splicing changes in muscles of *FRG1* mice

A peculiar distribution of affected muscles and the progressive character of the disease are among the features shared between FSHD patients and *FRG1* mice [25]. To investigate the molecular connections between *FRG1* over-expression and the impairment of muscle development and function, we took a genome-wide approach. Since FRG1 is thought to function in pre-mRNA splicing [28], [30], [32], [33], we employed splicing-sensitive microarrays [44], [45]. To identify changes that occur differently in muscles differentially affected in FSHD, we compared RNA extracted from *vastus lateralis* (highly affected) and *biceps brachii* (mildly affected) muscles of three *FRG1* mice and control littermates [25]. To follow disease progression, we analyzed 4-week-old animals (no signs of muscular dystrophy) and 13-week-old mice (fully developed muscular dystrophy) [25]. The full results for genes investigated at transcriptional and splicing levels are displayed in Tables S1 and S2, respectively.

**Figure 1:**
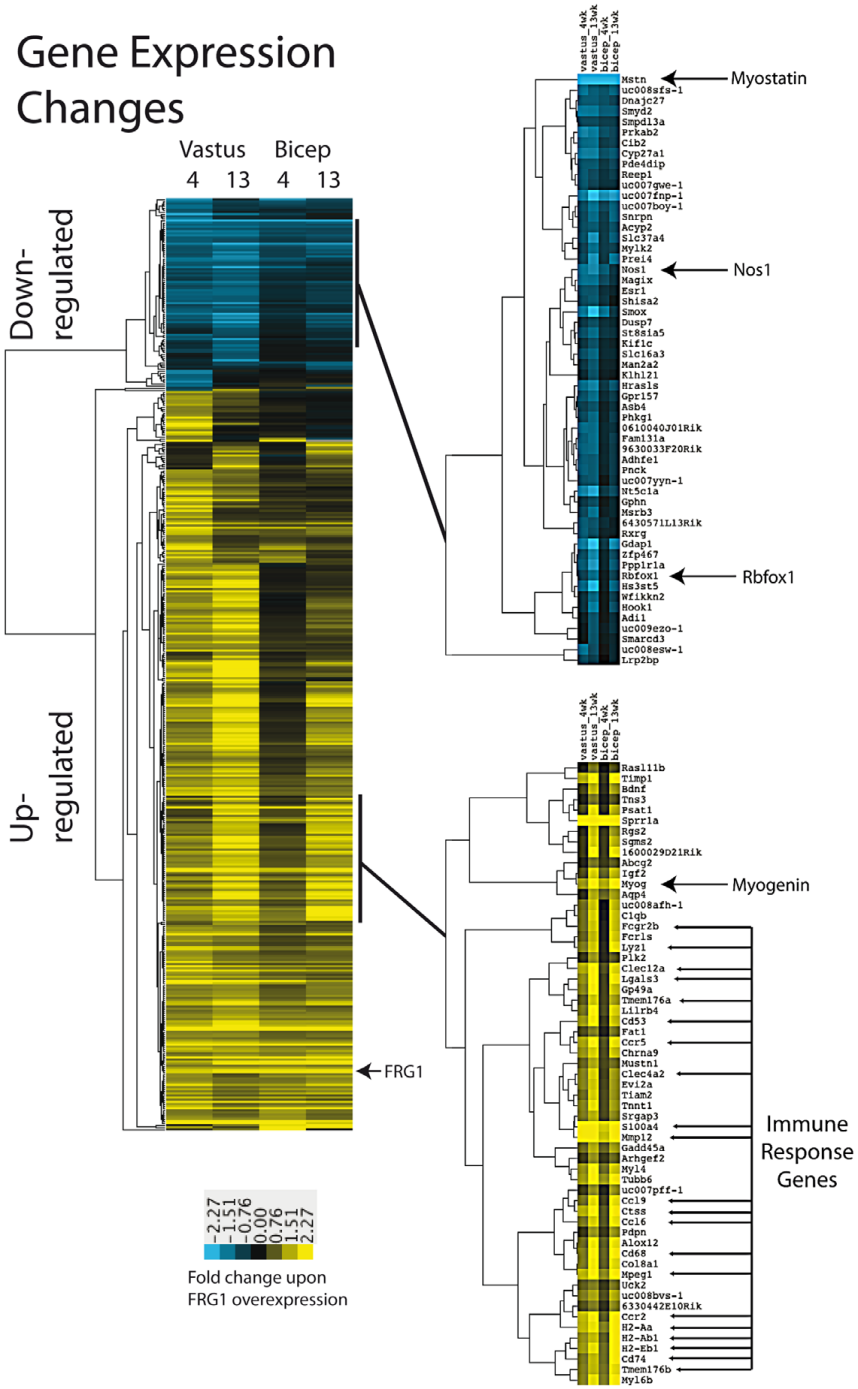
Gene expression changes in muscles of mice over-expressing FRG1. Average linkage clustering was applied to the fold change values of the 440 genes whose expression changes by at least 2 fold (SAM *q*<0.5) in at least one of the four comparison datasets. Selected nodes are expanded on the right. Genes with increased expression in FRG1 over-expressing muscles are shown in yellow in the heatmap, while those whose expression decreased are in blue.

We identified 440 genes whose expression changes more than 2 fold (q<0.05) in at least one of the four experiments (Table S3a). The effect of *FRG1* over-expression was very coherent among the four sets of samples. Indeed, all genes that changed did so in the same direction in every sample in which they changed. Upon hierarchical clustering (Figure 1), two groups of genes, those down-regulated and those up-regulated upon *FRG1* over-expression were clearly defined. No clear functional enrichment was observed upon GO analysis of the down-regulated genes, although there were clear signs of altered muscle development (see below). In contrast, up-regulated genes showed strong enrichment for inflammation and immune response genes (*e.g.* GO:0002376 P = 3.40E-05), as well as enrichment for contractile proteins. To directly assess the presence of an inflammatory infiltrate, we performed a specific histological staining using antibodies against the pan-hematopoietic marker CD45 and a quantitative analysis of CD45 positive cells by FACS. We found no evidence of inflammation in 4-week-old FRG1 mice (Figure S1). Hence, these results suggest that the muscle fibers are the source of the up-regulation of inflammatory genes identified by our microarray analyses performed in 4-week-old FRG1 mice. Among the genes affected by *FRG1* over-expression were several whose responses indicated developmental perturbations. The mRNA of *Myostatin*, an inhibitor of muscle development [46], was strongly down-regulated (7–10 fold). On the contrary, the mRNA of *Myogenin*, a promoter of muscle cell development [47], was strongly up regulated (3–7 fold). The mRNA of *Nos1*, a regulator of myogenic stem cells activation and differentiation frequently affected in muscular dystrophy [48], was down-regulated primarily in *vastus* (3 fold). We concluded that *FRG1* overexpression has a profound effect on the muscle transcriptome, including induction of immune response and inflammatory genes, and alteration of proper muscle development.

**Figure 2:**
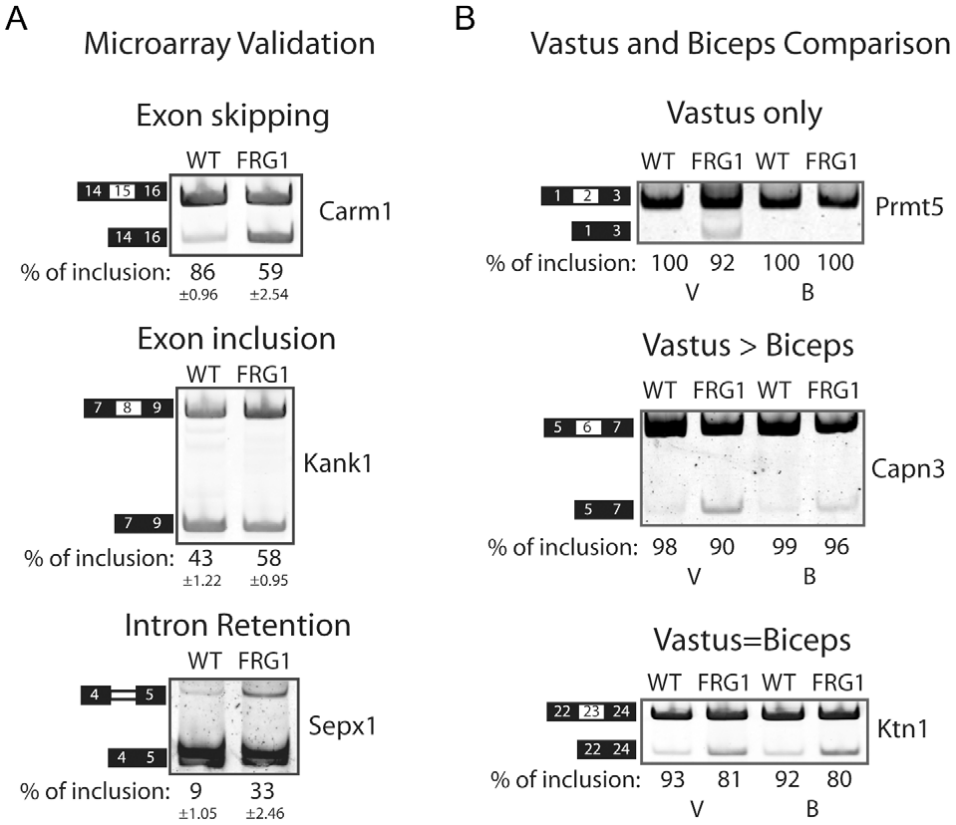
RT–PCR validation of splicing events belonging to different classes. (a) RT-PCR performed using RNA extracted from *vastus lateralis* muscle of *FRG1* and control littermates (n = 3 per group, 4 weeks old mice). Examples of increased exon skipping (Carm1), exon inclusion (Kank1) or intron retention (Sepx1) in *FRG1* mice is shown. RT-PCR products from three individual *FRG1* and control *WT* mice were quantified using the Typhoon and the skipping rates were calculated. Samples were judged as being different from *WT* if a t-test indicated that the sample was unlikely to be from the *WT* distribution with P<0.05. (b) Examples of alternative exons affected only (*Prmt5*), primarily (*Capn3*) or similarly (*Itga7*) in *FRG1* mice *vastus lateralis* compared to *biceps brachii* muscles. Numbers below gel images are the percentage of exon inclusion. Black boxes illustrate constitutive exons, white boxes alternatively spliced exons and double lines represent the affected intron.

We next searched the data for changes in alternative splicing. We observed 1735 splicing events whose include/skip isoform ratio changed significantly (|Sepscore|≥0.3; *p* = 0, see [49], [50]) between *FRG1* and wild type mice in *vastus lateralis* at 4 weeks of ages (V4w), 1005 events in *biceps brachii* at 4 weeks of ages (B4w), 1895 events *in vastus lateralis* at 13 weeks of ages (V13w) and 1454 events in *biceps brachii* at 13 weeks of ages (B13w) (Table S3b). Based on this simple count, more events were altered in the more affected muscle (vastus), and 13-week-old mice were more affected than 4-week-old mice. Among the transcripts identified were *Mtmr1* and *Tnnt3*, previously shown aberrantly spliced in FSHD [25]. We selected 48 genes for validation using semi-quantitative RT-PCR with RNA extracted from *vastus lateralis* muscle at 4 weeks of age from three new *FRG1* mice and control littermates that were not used for the microarray analysis (Table S4). An example of the typical results obtained for the different RNA splicing modes is displayed in Figure 2a. For the 48 genes, the microarray results were validated by RT-PCR in 83% of the cases (Figure S2), confirming that more genes are affected, and are more severely affected in *vastus lateralis* than in *biceps* (Figure 2b and Figure S3, Table S3b).

**Figure 3:**
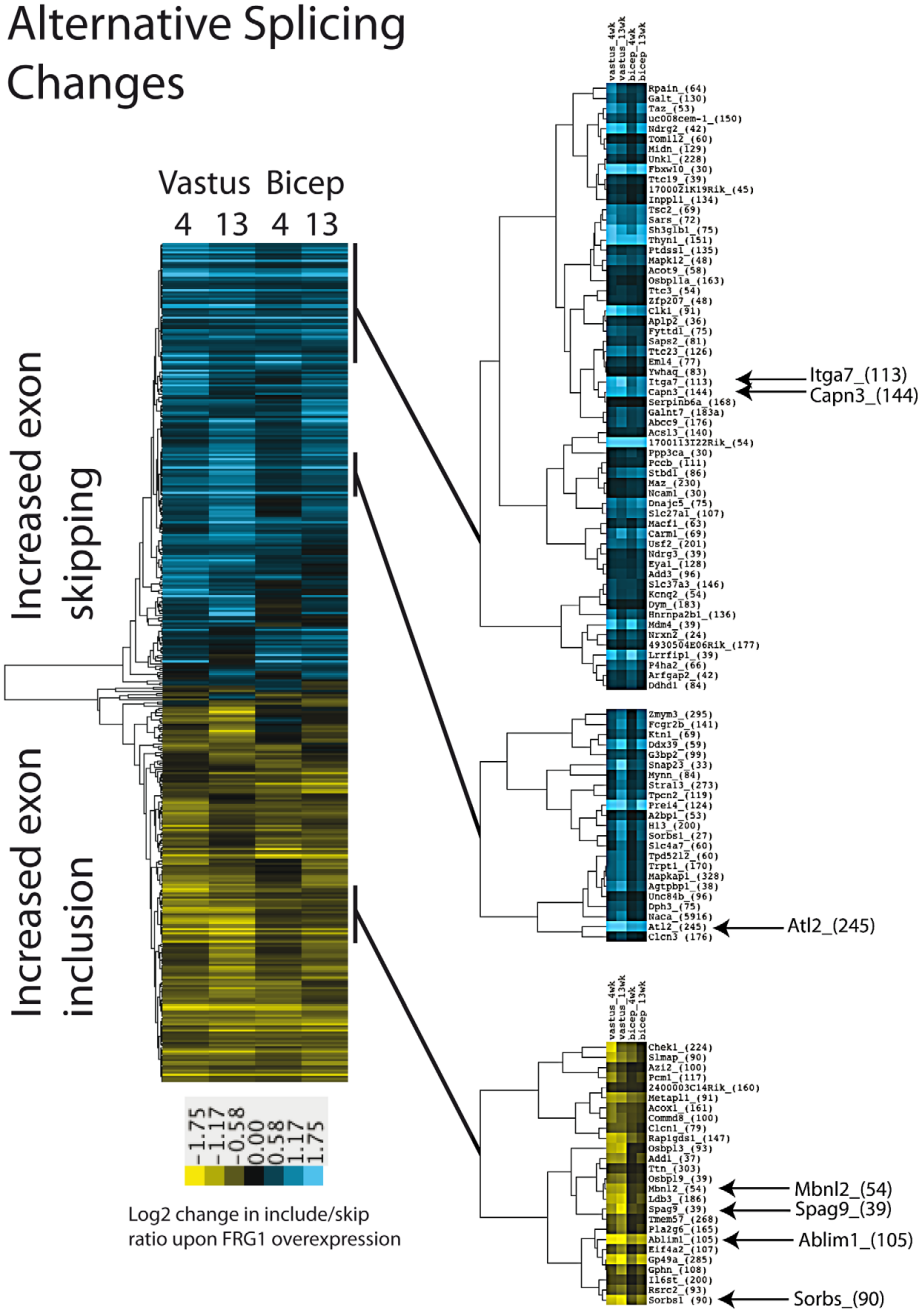
Alternative splicing changes in muscles of mice over-expressing FRG1. Average linkage clustering was applied to alternative cassette exon splicing events with |Sepscore|≥0.3 in at least one of the four comparison datasets (*q* = 0). Selected nodes are expanded on the right. Exons which shown increased inclusion in FRG1 over-expressing muscles are shown in yellow in the heatmap, while exons with decreased inclusion are represented in blue.

To study the pattern of splicing alterations in vastus and biceps muscles at 4- and 13- weeks, we clustered affected exons (q = 0, |Sepscore|≥0.3) by the log2 values of the ratio of inclusion/skipping ratios in *FRG1* tissues relative to wild type (Figure 3). Like the transcript level defects, the splicing defects were coherent, especially among strongly affected exons. Affected exons belong to genes involved in calcium regulation, muscle cell development and alternative splicing in muscle. For example, we noted increased inclusion of a 54 nucleotide exon in the mRNA for splicing factor Mbnl2 (Figure 3), however the significance of this change for the activity of Mbnl2 is not known. Our current lack of knowledge in general for the functional implications of most alternative splicing modifications makes it difficult to interpret the global impact for the changes identified by the microarrays.

To assess whether the observed splicing changes are associated with sequence motifs that might betray the splicing factor controlling them, we performed motif analysis.

**Figure 4:**
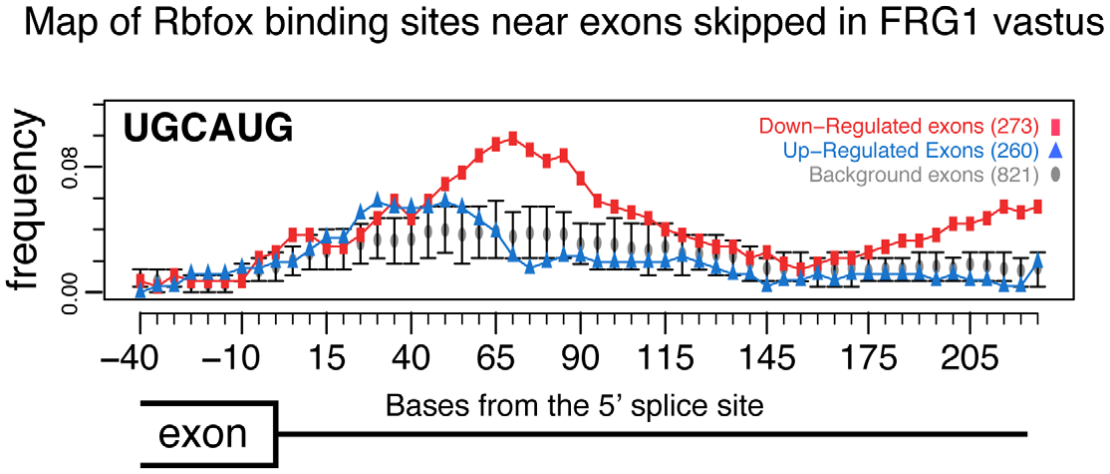
RNA map of Rbfox motifs downstream of exons. Each point represents the average frequency of UGCAUG in the 150 bp downstream of the 260 exons whose inclusion increases (blue triangles), the 273 exons whose inclusion decreases (red squares) or the 821 expressed alternative cassette exons whose splicing did not change in the comparison (gray circles, *q*>0.2 and |Sepscore|<0.3). Error bars indicate 95% confidence intervals of the mean frequency distribution for this population of background exons.

We counted the frequency of 6-mer “words” in the sequences within and surrounding exons whose inclusion was affected by *FRG1* over-expression. We found enrichment of TGCATG (P = 0.003), which is the highest ranking word in our search recognizably associated with a known RNA binding protein family, in this case, Rbfox [35]. This enrichment is found downstream of exons whose skipping increases in the *FRG1* muscles relative to wild type. To map the location of this motif in the exons that possess it, we measured the frequency of TGCATG in sliding windows across the exon set, as compared to the background frequency among exons which splicing did not change in the experiment (Figure 4). We observed a strong peak suggesting enrichment of the Rbfox binding site TGCATG in the region 70–90 nucleotides downstream from the 5′ splice site of exons whose inclusion decreased upon *FRG1* overexpression in 4-week-old vastus muscle. This location implicated one or more Rbfox family members in the activation of a set of exons normally expressed in *vastus* at this time. Furthermore this suggested that *FRG1* over-expression compromised function of one or more Rbfox family members. Consistent with this, we found that *Rbfox1* gene expression is down-regulated in tissues over-expressing *FRG1* (Figure 1).

### Splicing alterations are similar to those in *FRG1* over-expressing cultured myoblasts, but distinct from those in *mdx* mice

**Figure 5:**
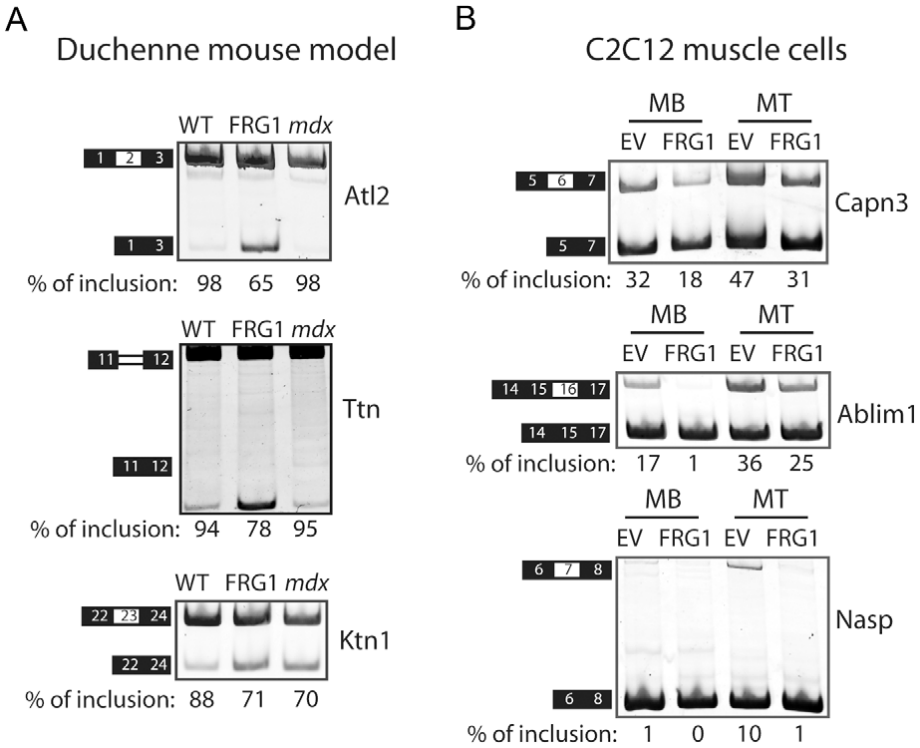
Alternative splicing changes are a primary consequence of *FRG1* overexpression. (a) Examples of alternative exons (*Atl2*) or introns (*Ttn*) spliced normally in the mouse model of Duchenne muscular dystrophy, *mdx* mice, and example of an alternative exon similarly altered in *FRG1* and *mdx* mice (*Ktn1*). (b) RT-PCR analysis of mRNA splicing variants from proliferating (MB) and differentiating (MT) C2C12 muscle cells over-expressing FRG1. Examples of alternative splicing changes present in both MB and MT (*Capn3*), only in MB (*Ablim1*), or only in MT (*Nasp*). Numbers are the percentage of exon inclusion. Black boxes illustrate constitutive exons, white boxes alternatively spliced exons and double lines represent the affected intron.

The altered splicing events identified here could be primary consequence of *FRG1* over-expression, secondary consequence of *FRG1* affecting expression of splicing factors for example, or far downstream consequences observable for any muscle disease. Most of the splicing changes were present in *FRG1* mice at 4 weeks (Table S3b), when the animals did not show signs of muscular dystrophy at histological and ultra-structural levels [25]. This strongly suggested that the altered splicing events were primarily associated with ongoing *FRG1* over-expression and related to early disease, before the appearance of changes due to muscle wasting. To assess this, we analyzed splicing in *mdx* mice, the mouse model of Duchenne muscular dystrophy [51], as an example of a muscle disease with a distinct genetic cause. This test showed that the vast majority (30 out of 40 events analyzed) of the events altered in *FRG1* overexpressing mice were spliced normally in *mdx* mice (Figure 5a and Figure S4). This excluded the possibility that most of the splicing alterations identified in *FRG1* mice were simply due to a common molecular phenotype found in all muscular dystrophies. To asses whether the phenotype observed in mouse tissues primarily concerns cell-independent functions or arises only in the presence of complex sets of cell types in developing tissues, we compared stable C2C12 muscle cells expressing either the empty vector (C2C12-*EV*) or *FRG1* at low levels (C2C12-*FRG1*) ([25] and Figure S5). We analyzed both proliferating (myoblast, MB) and differentiating (myotubes, MT) cells. For the majority of the genes tested, we found that C2C12- *FRG1* cells displayed splicing alterations similar to *FRG1* mice (Figure 5b and Figure S5). Collectively, these findings indicated that *FRG1* over-expression induced a characteristic set of splicing changes that can be observed in both cultured muscle cells and in tissues, and is distinct from that observed in a different model of muscular dystrophy.

### The alternative splicing factor *Rbfox1* is selectively down-regulated in mice and cells over-expressing *FRG1* and in FSHD patients

A recent report indicated that recombinant FRG1 binds RNA *in vitro* and is associated to *FXR1* and *FRG1* mRNAs [52]. If FRG1 is an RNA binding protein, affected splicing events could be directly controlled by FRG1. Using the same antibodies and experimental conditions to perform RNA immunoprecipitation (RIP) as reported [52], we could not detect specific FRG1 association with the set of RNAs whose splicing is altered in *FRG1* over-expressing C2C12 cells (Figure S6a). FRG1 RIP was also performed using an alternative protocol that we have successfully applied to other RNA-binding proteins (see below), with the same result (Figure S6b).

**Figure 6:**
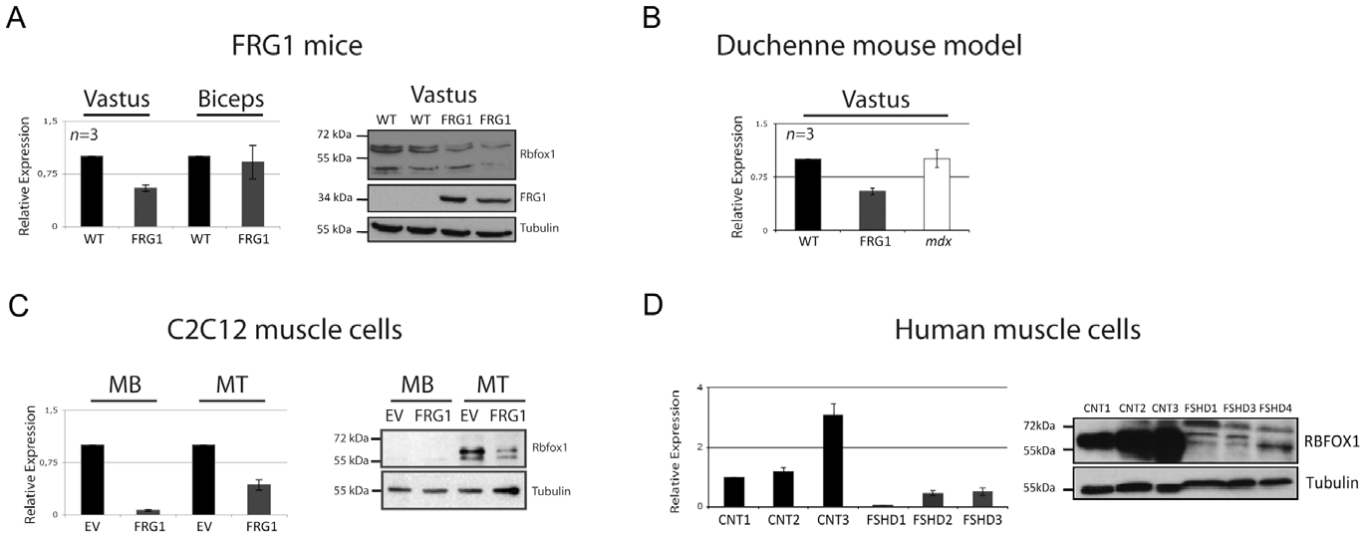
*Rbfox1* is selectively down-regulated in mice, in cells over-expressing *FRG1*, and in FSHD patients. (a) Left panel: real-time RT-PCR analysis showing that in *FRG1* mice *Rbfox1* expression is preferentially downregulated in *vastus lateralis* compared to *biceps brachii*. *Gapdh* expression was used for sample normalization. Right panel: immunoblotting on *vastus lateralis* using anti-Rbfox1 antibody. Tubulin was used as loading control and FRG1 antibody as confirmation of the genotype. (b) Real-time RT-PCR analysis showing that *Rbfox1* expression is normal in *mdx* mice. (c) Left: real-time RT-PCR analysis showing that *Rbfox1* expression is down-regulated in proliferating (MB) or differentiating (MT) C2C12 muscle cells over-expressing *FRG1*. Right: immunoblotting using protein extracted from the same samples as in (c, left) using anti-Rbfox1 antibodies. Tubulin was used as loading control. Note that in proliferating C2C12 cells Rbfox1 protein is almost undetectable, while its levels significantly increase during myogenic differentiation. (d) Left panel: real-time RT-PCR analysis of *RBFOX1* expression using RNA extracted from muscle cells derived from three different normal individuals and three different FSHD patients. Right panel: immunoblotting using protein extracted from the same samples as in (d, left) using anti-RBFOX1 antibody. Tubulin was used as loading control.

Since we did not find evidence of FRG1 binding to the RNAs whose splicing is altered in FRG1 mice, we investigated the hypothesis that FRG1 might alter splicing through its effect on the activity or the expression of known splicing factors. Since *FRG1* over-expression appeared to down-regulate *Rbfox1* mRNA and inhibited the activation of exons with Rbfox1 binding sites, we decided to examine more carefully the expression of *Rbfox* splicing factors upon *FRG1* over-expression. While *Rbfox2* and *Rbfox3* expression was not significantly affected (data not shown), expression of Rbfox1 protein was significantly reduced in *vastus lateralis* of *FRG1* mice (Figure 6a). Furthermore, the extent of down-regulation of *Rbfox1* expression paralleled the severity of the disease in different muscles (Figure 6a). In addition, *Rbfox1* was expressed at normal levels in *mdx* mice, indicating that its down-regulation in *FRG1* mice is not generally associated with muscular dystrophy (Figure 6b). *Rbfox1* mRNA down-regulation was also displayed by both proliferating (MB) and differentiating (MT) C2C12-*FRG1* muscle cells, indicating that it is a cellular consequence of *FRG1* over-expression (Figure 6c, left). Rbfox1 protein down-regulation was evident in differentiating (MT) C2C12-*FRG1* muscle cells, while the expression level of Rbfox1 in proliferating (MB) C2C12-*FRG1* muscle cells was below the sensitivity of our immunoblotting (Figure 6c, right). Importantly, *RBFOX1* mRNA and RBFOX1 protein were down-regulated in primary human muscle cells derived from FSHD patients (Figure 6d).

**Figure 7:**
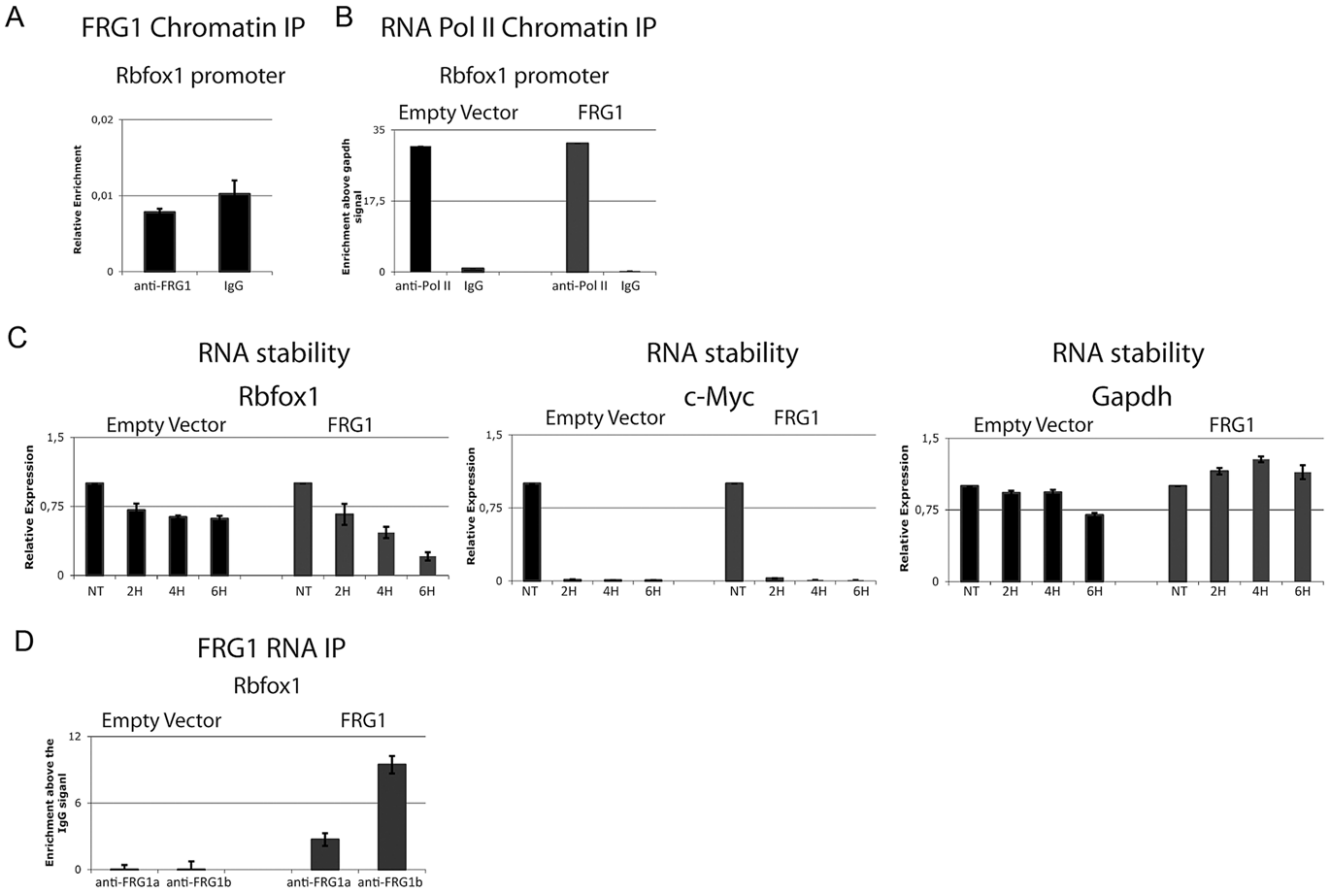
FRG1 regulates the stability of the *Rbfox1* mRNA. (a) ChIP of FRG1 in the promoter region of the *Rbfox1* gene. (b) The distribution of RNA pol II on C2C12-*EV* and C2C12-*FRG1* cells in the promoter region of the *Rbfox1* gene. (c) Real-time RT-PCR of the kinetics of *Rbfox1*, *c-Myc* and *Gapdh* expression after 8 hours of ActD (Actinomycin D) treatment on C2C12-*EV* and C2C12-*FRG1* cells. (d) RNA-IP experiment on samples from (c) using anti-FRG1 antibodies or control IgG antibodies. Immunoprecipitated material was analyzed by real-time RT-PCR, normalized versus the relative input and plotted as fold enrichment versus the IgG. RT-minus control experiments showed the absence of DNA contamination (data not shown).

*FRG1* over-expression could down-regulate *Rbfox1* expression at transcriptional or post-transcriptional level. To investigate if FRG1 regulates *Rbfox1* at the transcriptional level, we performed chromatin immunoprecipitation (ChIP) using anti-FRG1 antibodies. We found no evidence of FRG1 binding to the promoter of the *Rbfox1* gene (Figure 7a). To further investigate this, we performed ChIP using anti-RNA pol II antibodies, as it is known that RNA pol II recruitment correlates with the transcriptional rate [53]. As shown in Figure 7b, we found no change in RNA pol II loading on the *Rbfox1* promoter in *FRG1* over-expressing cells compared to control. Collectively, these results indicate that FRG1 does not regulates *Rbfox1* expression at the transcriptional level and suggest that it could act at post-transcriptional level. To test this, we monitored the stability of the *Rbfox1* mRNA upon *FRG1* over-expression. As shown in Figure 7c, the stability of the *Rbfox1* mRNA was greatly reduced in *FRG1* over-expressing cells compared to control. The result appears specific since we found no change in the stability of two other mRNAs: *c-Myc* and *Gapdh*. To investigate if *Rbfox1* could be a direct FRG1 target, we performed RIP following UV-crosslinking. As shown in Figure 7d, using two different anti-FRG1 antibodies [52], we found an FRG1 association to the *Rbfox1* mRNA selectively in cells over-expressing FRG1. Altogether, our results strongly suggest that FRG1 down-regulates *Rbfox1* by decreasing the stability of its mRNA.

### Identification of direct Rbfox1 targets altered in *FRG1* mice

**Figure 8:**
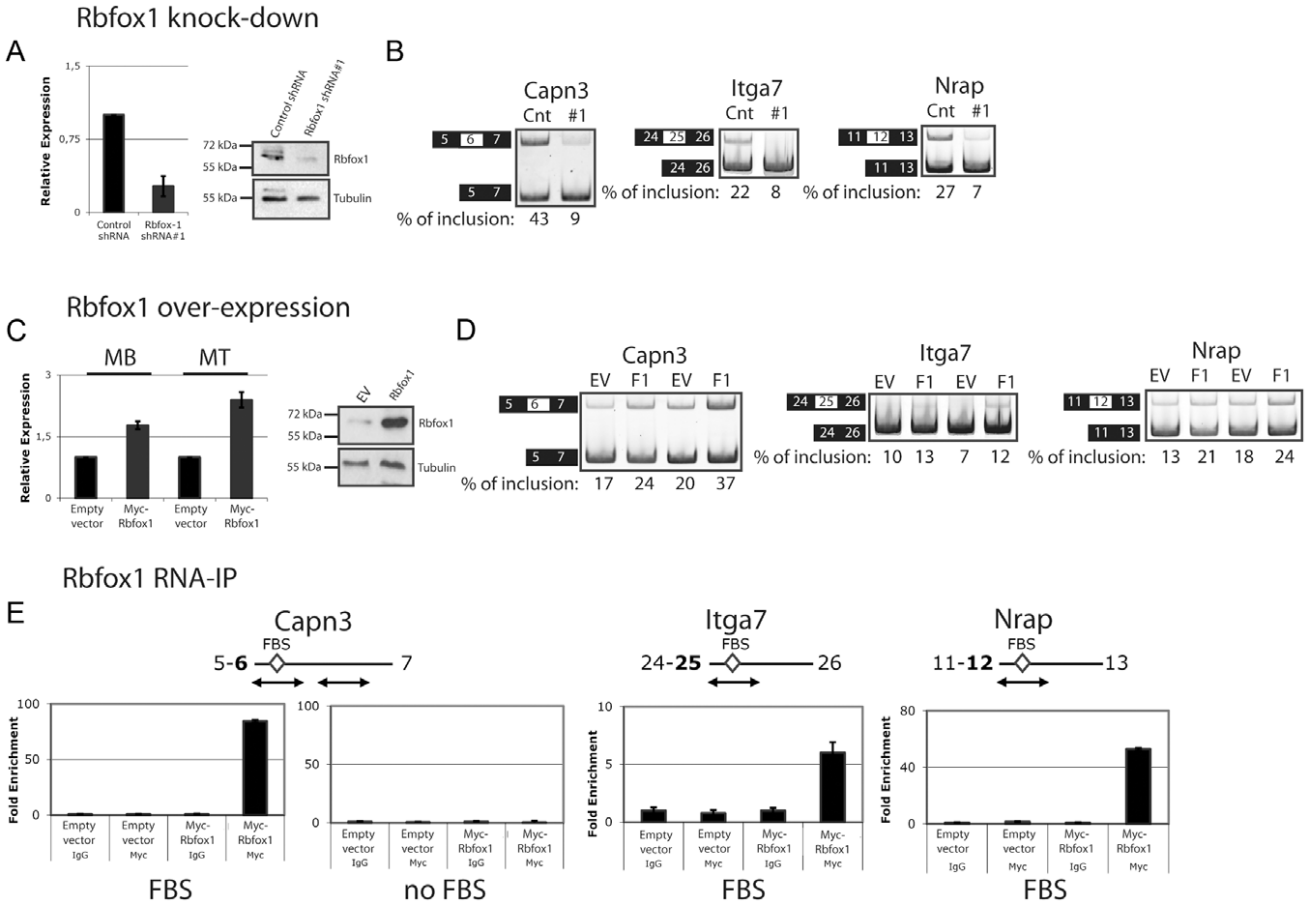
*Rbfox1* down-regulation is responsible for significant portion of the splicing alterations in *FRG1* mice. (a) Specific *Rbfox1* knockdown was confirmed by real-time RT-PCR and immunoblotting using RNAs and proteins isolated from C2C12 muscle cells expressing a control non-silencing shRNA or an shRNA specific for *Rbfox1* (shRNA#1). (b) Examples of alternative splicing changes caused by *Rbfox1* knockdown are showed. Numbers are the percentage of exon inclusion. Black boxes illustrate constitutive exons, white boxes alternatively spliced exons. (c) *Rbfox1* overexpression causes alternative splicing changes opposite to *FRG1* over-expression. Specific *Rbfox1* over-expression was confirmed by real-time RT-PCR and immunoblotting using RNAs and proteins isolated from C2C12 muscle cells expressing an empty vector (*EV*) or a Myc-tagged Rbfox1 (*F1*) either in proliferating or differentiating C2C12 muscle cells. (d) Examples of alternative splicing changes caused by *Rbfox1* over-expression are showed. Black boxes illustrate constitutive exons, white boxes alternatively spliced exons. (e) Selective *in vivo* association of Rbfox1 to target regions displaying putative Fox binding sites (FBS). RIP experiment on samples from (c) using anti-Myc or control IgG antibodies. Immunoprecipitated material was analyzed by RT-PCR, quantified using the Typhoon, normalized versus the relative input and plotted as fold enrichment versus the IgG. RT-minus control experiments showed the absence of DNA contamination (data not shown).

To test directly whether Rbfox1 regulated splicing of the exons we identified in *FRG1* over-expressing cells and tissues, we performed *Rbfox1* knockdown and overexpression studies in C2C12 cells expressing normal levels of *FRG1*. We selected two independent shRNAs (*Rbfox1* shRNA #1 and #2) that significantly decreased *Rbfox1* expression in C2C12 muscle cells at both its mRNA and protein levels (Figure 8a and Figure S7b). For all pre-mRNAs displaying putative Fox binding sites (FBS) analyzed (6/6), *Rbfox1* knockdown caused alternative splicing changes similar to those detected in C2C12-*FRG1* (Figure 8b and Figure S7a and S7c). None of the pre-mRNA lacking putative FBS analyzed (5/5) displayed altered splicing upon *Rbfox1* knockdown (Figure S7a and S7c). The directions of the alternative splicing changes caused by *Rbfox1* knockdown agrees with the positions of the putative FBS as previously noted [35], see Figure 8b, Figure S7a and S7c).

Next, we analyzed alternative splicing in C2C12 cells over-expressing *Rbfox1* (Figure 8c). In this case, for all pre-mRNAs analyzed (6/6) *Rbfox1* over-expression caused alternative splicing changes opposite to those detected in C2C12-*FRG1* or *Rbfox1* knockdown C2C12 cells, and these also agree with the position of the putative FBS (Figure 8d, Figure S7d). To determine if Rbfox1 regulated pre-mRNAs were bound by Rbfox1 *in vivo* we performed RIP experiments. As shown in Figure 8e and Figure S7e, Rbfox1 was associated with all pre-mRNAs displaying the putative FBS by RIP. No Rbfox1 RIP signal was found for a region of the *Calpain 3* transcript lacking a putative FBS (Figure 8e) or to the control *Gapdh* transcript (Figure S7e) suggesting that the tested pre-mRNAs were direct Rbfox1 targets. Collectively, these results strongly suggest that *Rbfox1* down-regulation is responsible for a subset of the alternative splicing changes observed in cells and muscles engaged in the *FRG1* overexpression associated with FSHD.

### Altered *Calpain 3* splicing in FSHD patients

**Figure 9:**
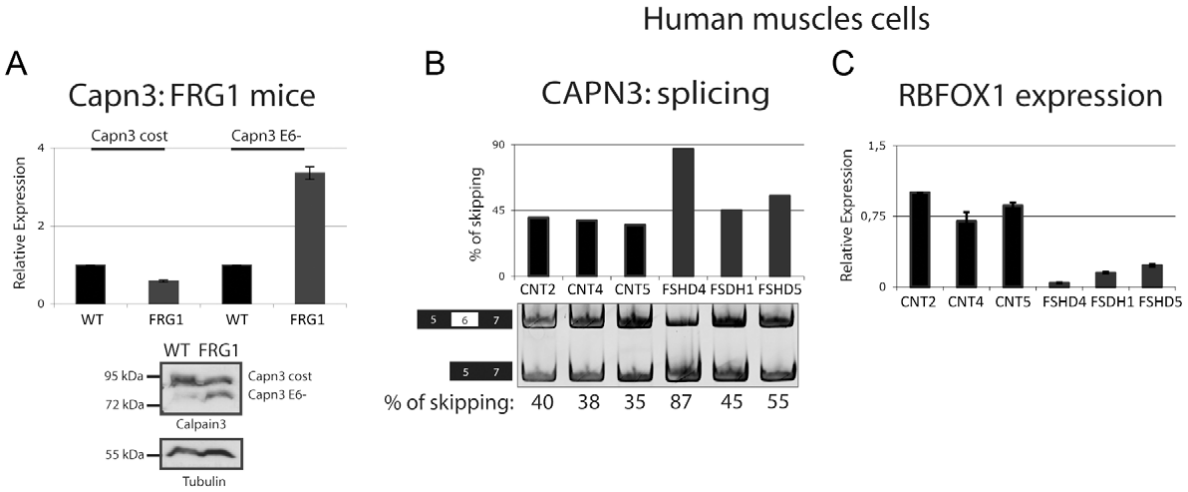
Alternative splicing isoform of *Calpain 3* increased in *FRG1* mice and in FSHD patients. (a) Real-time RT-PCR and immunoblotting analysis confirming that the *Capn3* alternative splicing isoform lacking exon 6 (*Capn3 E6-*) is increased in the *vastus lateralis* muscle from *FRG1* mice. (b) RT-PCR analysis of *CAPN3* splicing in human muscle cells derived from three different healthy subjects and three different FSHD patients indicates increased expression of *CAPN3 E6-* isoform in FSHD patients. Numbers below the image are the percentage of exon skipping. Black boxes illustrate constitutive exons, white boxes alternatively spliced exon. RT-PCR products were quantified using the Typhoon. (c) *RBFOX1* expression analysis was performed on RNA extracted from the same samples as in (b) by real-time RT-PCR.

Among the pre-mRNAs displaying aberrant splicing as a result of *FRG1* overexpression in muscles (Figure 3), or after *Rbfox1* knockdown and *Rbfox1* overexpression in cultured cells (Figure 8), we focused our attention on *Calpain 3* (*Capn3*). *Capn3* encodes for a muscle-specific Calcium-dependent protease [50] and its mutation is responsible for limb-girdle muscular dystrophy type 2A (LGMD2A) [49]. A *Capn3* alternative splicing isoform lack exon 6 (*Capn3 E6–*) was increased in *FRG1* mice (Figure 3). Increased *Capn3 E6–* upon *FRG1* over-expression was confirmed by semi-quantitative RT-PCR (Figure 2b and Figure 5b), real-time RT-PCR and immunoblotting (Figure 9a). Notably, increased *CAPN3 E6-* expression was observed in FSHD muscle cells (Figure 9b) that also displayed *Rbfox1* down-regulation (Figure 9c).

Transgenic mice with muscle-specific increased *Capn3 E6–* expression display some similarity to *FRG1* mice [25], [54]. For example, both models display kyphosis, reduced muscle mass, reduced muscle fiber cross-sectional area and increased number of centrally nucleated muscle fibers [25], [54]. Moreover, in both models *vastus lateralis* is more affected than *biceps brachii* [25], [54]. Finally, Evans blue dye staining and creatine kinase levels are normal in *Capn3 E6–* and *FRG1* mice indicating that muscle disease in both models, like in FSHD patients, do not compromise sarcolemma integrity [25], [54]. Based on this, it is tempting to speculate that an increase in *Capn3 E6–* isoform expression as a consequence of *FRG1*-mediated down regulation of *Rbfox1* could contribute to FSHD.

### *FRG1* over-expression, *Rbfox1* knockdown of *Capn3 E6–* all inhibit myogenic differentiation

**Figure 10:**
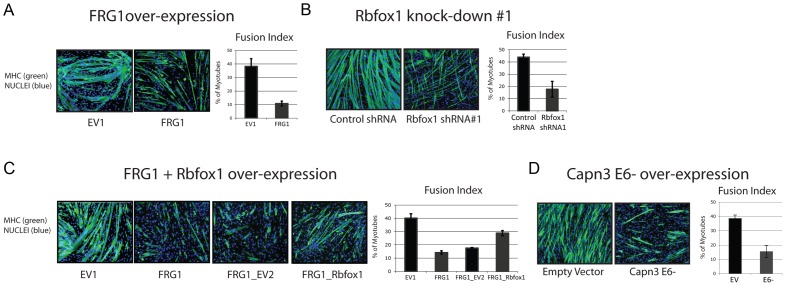
Altered *Calpain 3* splicing inhibits myogenesis. (a) C2C12 muscle cells over-expressing *FRG1*, (b) *Rbfox1* knockdown with shRNA#1 and (d) over-expressing *Capn3 E6-* display a reduced myogenic differentiation. (c) C2C12 muscle cells over-expressing *FRG1* and *Rbfox1* present increase myogenic differentiation compared to C2C12-*FRG1*. Left: immunofluorescence using antibodies specific for skeletal muscle myosin heavy chain (MHC), a typical marker of terminally differentiated muscle cells. Right: fusion index defined as the percentage of nuclei belonging to MHC-positive cells with three or more nuclei. The values reported in the graph are the means plus-minus standard deviations from two separate experiments performed in triplicate.

Several reports indicate a muscle differentiation defect in FSHD [5]–[7], [9]–[11]. To investigate whether this phenotype might be mediated by *FRG1* over-expression, we compared the differentiation capability of C2C12 muscle cells over-expressing *FRG1* (*FRG1*) or the empty vector (EV1). As shown in Figure 10a, *FRG1* over-expression significantly reduced the differentiation capability of C2C12 cells. Quantification of the fusion index showed that *FRG1* cells form only 25% of the myotubes formed by *EV1* (Figure 10a). C2C12 cells expressing two independent shRNAs specific for *Rbfox1* (shRNA#1 and #2) also displayed a reduction in myogenesis, although lower compared to *FRG1* cells (compare Figure S7b, Figure 10a and Figure 10b).

FRG1 and Rbfox1 might act independently on muscle differentiation. To establish a link between the two proteins, we performed rescue experiments by over-expressing *Rbfox1* in *FRG1* over-expressing cells. By performing differentiation experiments, we observed a partial amelioration of the phenotype in *Rbfox1/FRG1* over-expressing cells compared to control cell lines (Figure 10c). This result strongly suggests that Rbfox1 plays an important role in the myogenic inhibition caused by FRG1 over-expression.

Importantly, a differentiation defect was also displayed in C2C12 muscle cells selectively over-expressing *Capn3 E6–* (Figure S8 and Figure 10d). Thus, as with the mouse models, the C2C12 cell system suggested that an increase in *Capn3 E6–* isoform expression as a consequence of *FRG1*-mediated down-regulation of *Rbfox1* could contribute to FSHD.

## Discussion

FSHD is a complex disease in which the severity, rate of progression and distribution of muscle weakness display great variability even among close family relatives. The unusual nature of the mutation that causes FSHD and its complex effect on chromatin surrounding the 4q35 region makes it highly unlikely that the root cause can be attributed to a single gene. Two 4q35 genes have been investigated as candidate FSHD genes, *DUX4* and *FRG1* [23], [55], [56]. Individually, each gene causes myopathic changes when over-expressed in muscle cells *in vivo* [25], [57]. For Dux4, a disruption of muscle development/differentiation through competition with Pax3/Pax7 myogenic regulators has been reported [58]. Our gene expression profiling of *FRG1* mice (Figure 1) and the myogenic defects caused by *FRG1* over-expression in C2C12 cells (Figure 10) indicate that FRG1 also disrupts muscle differentiation. Surprisingly, we found that a significant number of genes involved in inflammation and immune response are up-regulated in vastus muscles of *FRG1* mice at 4 weeks of age (Figure 1). This is particularly intriguing since at this age *FRG1* mice do not show any evidence of infiltrate/inflammation by histology, immunohistochemistry with anti-CD45 (expressed on all hematopoietic cells, except erythrocytes and plasma cells) antibodies or by FACS analysis of single cell preparations from the muscle (Figure S1). Moreover, at 4 weeks of age there is no sign of necrosis or muscle regeneration in *FRG1* mice (i.e. no increase in centrally nucleated muscle fibers) (Xynos et al., *submitted*). Hence, it appears that the increased expression of inflammation and immune response genes originates directly from the muscle cells themselves. In classical muscular dystrophies, inflammation is secondary to the necrosis of muscle fibers. On the contrary, our results suggest that in *FRG1* mice recruitment of hematopoietic cells is induced by the muscle directly expressing inflammation and immune response genes, before the appearance of muscle fiber necrosis and regeneration. This is in agreement with the fact that in FSHD, unlike classical muscular dystrophies, the inflammation is not simply an event secondary to muscle wasting but takes place early on in the disease and could triggers the disease process [59]–[62]. Based on this, it is tempting to speculate that a spurious activation of inflammation and immune response genes within muscle cells by FRG1 (and possibly DUX4, [63]) could play a role in FSHD onset and progression.

While the specific function of FRG1 is still unknown, an increasing body of evidence suggests a role in alternative splicing regulation [25], [28]–[30], [32], [33]. In this study, we investigated this by a genome-wide accounting of alternative splicing events affected by *FRG1* over-expression. In both *FRG1* mice and in FSHD patients, different muscles are affected differentially, the disease worsens with time [25], and the splicing profiles of differentially affected muscles of *FRG1* mice at different ages correlates with the severity of the disease (Table S3b). Importantly, most of these splicing alterations are already evident in pre-symptomatic *FRG1* mice, suggesting that they are related to disease onset and early progression (Figure 2). Moreover, comparisons with the splicing profile of the mouse model of Duchenne muscular dystrophy strongly indicate that the identified splicing changes are specific for the FSHD model rather than as a signature of diseased muscle (Figure 5a). Finally, most of the splicing alterations also occur in C2C12 muscle cells over-expressing *FRG1* (Figure 5b), indicating a fundamental cellular response rather than a complex and secondary tissue effect. Collectively, these data identify a large set of splicing alterations that are a primary consequence of *FRG1* over-expression and are involved in disease onset.

Our results suggest that the exons identified by the splicing-sensitive arrays are not direct FRG1 targets and that FRG1 function at least in part by regulating the expression of the splicing factor Rbfox1 at post-transcriptional level. We found that the Rbfox1 recognition sequence is over-represented in the alternative splicing events altered in *FRG1* mice (Figure 4) and by *Rbfox1* knockdown, over-expression and RIP experiments we confirmed that these are direct Rbfox1 targets (Figure 8 and Figure S7). *Rbfox1* expression is down-regulated in *FRG1* mice (Figure 6a), C2C12 cells over-expressing *FRG1* (Figure 6c), and in FSHD patients (Figure 6d). We also found that the level of *Rbfox1* down-regulation parallels the severity of the disease in different muscles of *FRG1* mice, while it is expressed at normal levels in the mouse model of Duchenne muscular dystrophy (Figure 6b). Importantly, our results suggest that the *FRG1* over-expression down-regulates the expression of *Rbfox1* by decreasing the stability of its mRNA (Figure 7). Collectively, these results support a role for altered *Rbfox1* expression in FSHD. While FSHD is primarily a disease of the skeletal muscle, epilepsy, mental retardation and autism have also been described in severely affected patients [12]–[15]. Since the same symptoms are caused by *Rbfox1* mutation or down-regulation [64]–[73], it is tempting to speculate that *Rbfox1* down-regulation could be involved in the neurological manifestations of FSHD. Among the transcripts displaying FRG1 and Rbfox1 dependent aberrant splicing, we focused on *Capn3*, as mutations in *Capn3* cause LGMD2A [49], and FSHD and LGMD share clinical features [74], [75]. The clinical spectrum of FSHD can include prominent pelvic girdle weakness and, in some individuals, only minimal facial muscle involvement, leading to confusion in diagnosis [74]. Indeed, it has been reported that ∼8% of patients with a diagnosis of LGMD may in fact have FSHD [75].

We found that *FRG1* over-expression is associated with increased expression of a *Capn3* splicing isoform lacking exon 6 (*Capn3 E6-*) in both *FRG1* mice and FSHD patients (Figure 2 and Figure 9b). Interestingly, transgenic mice over-expressing *Capn3 E6-* display phenotypic similarities to *FRG1* mice [25], [54]. The fact that *Capn3 E6-* is already increased in pre-symptomatic *FRG1* mice (Figure 2b and Figure 9a) and that its expression is normal in the mouse model of Duchenne muscular dystrophy (Figure S4) suggests that altered *Capn3* splicing is a primary consequence of *FRG1* overexpression and is not simply secondary to muscle wasting. This is confirmed by the fact that *FRG1* over-expression in tissue culture is sufficient to drive increased *Capn3E6-* expression (Figure 5b). Intriguingly, we found that *Capn3 E6-* expression correlates with the degree of muscular dystrophy in different muscles and at different ages (Figure 2 and Figure 3) suggesting that altered *Capn3 E6-* could explain the differential susceptibility of different muscles to *FRG1* over-expression and the progression of the disease over time.

Capn3 belongs to the Calpain family of non-lysosomal, soluble cytosolic calcium-dependent proteases [76]. *Capn3* is highly expressed in the skeletal muscle and numerous data suggest a role for Capn3 in the maintenance of muscle integrity and function [76]. Capn3 presents three insertion sequences that differentiate it from all other calpains and might contribute to its muscle-specific functions. These regions, called NS, IS1, and IS2 are encoded by exons characterized by developmentally regulated alternative splicing [77]. Exon 6 encodes for IS1, a Capn3 domain involved in auto-inhibitory function through autolytic cleavage [76]. *Capn3 E6-*, encoding the isoform of Capn3 lacking IS1, is not expressed in healthy adult muscle and appears to be primarily important during development and muscle regeneration [76]. Accordingly, *Capn3 E6-* over-expression in adult muscle results in muscles that appear developmentally abnormal, underlining the importance of this alternative splicing event [54]. It has been suggested that alternative splicing isoforms of *Capn3* could serve specific roles in regulating muscle differentiation [54]. Intriguingly, we found that *Capn3 E6-* over-expression in C2C12 muscle cells is sufficient to cause a differentiation defect similar to *FRG1* over-expression or *Rbfox1* down-regulation (Figure 10). Since myogenic defects have been reported in FSHD [5]–[11], it is tempting to speculate that altered *Capn3* splicing could contribute to the pathogenesis of FSHD.

In conclusion, our studies have provided insight on the molecular pathogenesis of one of the most important muscle diseases. Moreover, our findings advance the understanding of the alternative splicing regulation of myogenic differentiation and identify possible therapeutic targets for the treatment of FSHD.

## Materials and Methods

### Ethics statement

All procedures involving human samples were approved by the Fondazione San Raffaele del Monte Tabor Ethical Committee. All animal procedures were approved by the Institutional Animal Care and Use Committee of the Fondazione San Raffaele del Monte Tabor and were communicated to the Ministry of Health and local authorities according to Italian law.

### Human samples

Human primary myoblasts were obtained from the Telethon BioBank of the C. Besta Neurological Institute, Milano, Italy.

### Animals and tissue preparation

All mice were maintained in Specific Pathogen Free conditions. *FRG1-high* transgenic mice were described previously [25]. *mdx* mice were purchased from the Jackson laboratory (Bar Harbor, ME, USA). Skeletal muscle from both *vastus lateralis* and *biceps brachii* were carefully dissected from individual age- and sex-matched mice and maintained in RNA later (Ambion). RNA was isolated using RNeasy Fibrous Tissue Midi or Mini Kit (Qiagen) according to the manufacturer's instructions. All RNA samples were treated with DNase I. Concentration, DNA-free RNA purity and integrity was determined by Nanodrop and by Agilent 2100 Bioanalyzer. Only samples with RNA integrity number ≥8 were used.

### Splicing-sensitive microarrays

Ribosomal RNAs were removed from samples using the RiboMinus Transcriptome Isolation Kit (Invitrogen) according to the manufacturer's instructions. An amplified, biotinylated complementary DNA target was produced using the GeneChip Whole Transcript Sense Target Labeling and Control Reagents kit (Affymetrix) according to the manufacturer's instructions. Each sample target was hybridized overnight to a Mouse GeneSplice Array (Affymetrix PN 540092). Hybridized arrays were processed using the Affymetrix Fluidics Station 450 and scanned with an Affymetrix GeneChip scanner. Microarray data have been deposited in GEO (http://www.ncbi.nlm.nih.gov/geo/) under accession number GSE32073. Sepscore-based alternative splicing analysis was performed as previously described [45]. Expression was estimated according to probe sets that measure constitutive features for each gene. Differentially expressed genes were identified using SAM (Significance Analysis of Microarrays) [78]. Hierarchical clustering was done using Cluster [79] and visualized using Java Treeview [80].

### Sequence motif analysis

“Word counting” was performed by counting the number of cassette exons containing a specific 6mer within a window of sequence (*e.g.* 150 nucleotides of flanking downstream intron). The difference in counts of sequences containing the motif of interest from the test and background sets of exons are tested for significance by Fisher's exact test.

We identified areas enriched in the TGCATG motif by sliding a window of 50 nucleotides in 5-nucleotide steps over portions of the intron flanking the alternative exon. Specifically, we examined the area starting at the 5′ splice site and extending 150 nucleotides downstream. At each step, we contrasted the number of motifs observed for each of three groups of alternative splicing events: those exons repressed in 4 week *vastus* (Sepscore<0.3, *q* = 0), those induced at 4 weeks (Sepscore>0.3, *q* = 0), and background events that were from genes expressed in 4 week *vastus* but that showed no significant change in splicing (|Sepscore|<0.3, p>0.2). For the repressed and induced cassette exons, we plotted the motif frequency within the window: the total number of motifs observed divided by the number of events. We then randomly selected a set of background events equal in size to the smaller of the set of repressed or induced events and measured the motif frequency. From 100 such samplings, we estimated the average background frequency (plotted as points), and the 95% confidence intervals (plotted as error bars).

### Cell culture, myogenic differentiation, and RNA extraction

C2C12 muscle cells and 293T cells were obtained from the ATCC - LGC Standards (Sesto San Giovanni, MI, Italy) were grown in DMEM (EuroClone) supplemented with 10% FBS (EuroClone) and 1% antibiotic at 37°C in 5% CO2 and 5% O2. To induce muscle differentiation, C2C12 muscle cells were growth to near confluence and then switched to DMEM 2% horse serum for three days (EuroClone). Fresh differentiation medium was changed every day. To determine the extent of myogenic differentiation, differentiating C2C12 cells were fixed with 4% of paraformaldehyde and stained with antibodies against the differentiation marker myosin heavy chain (MHC) (mouse monoclonal, 1∶2, #MF20, Developmental Studies Hybridoma Bank, University of Iowa) according to manufacturer's instructions. At least 1,500 nuclei from MHC-positive cells were counted from several random fields. The fusion index was calculated as follows: (MHC-stained myocytes containing >2 nuclei/total number of nuclei)×100. All experiments were performed in triplicate. RNA from C2C12 muscle cells was extracted using Trizol and PureLink RNA kit (Invitrogen) according to manufacturer's instructions.

### RT–PCR validation of microarray results

cDNA was generated using SuperScript III First-strand synthesis supermix for qRTPCR (Invitrogen). 25 ng of cDNA were used as a template for PCR with each primer pair (designed with Primer3 software; Table S5). PCR reactions were carried out using Go Taq Polymerase (Promega), were separated on native TBE acrylamide gels and stained with SYBER Gold (Invitrogen). Gels were imaged and signals quantified with a Typhoon FLA 9000 Biomolecular Imager. We quantified the RT-PCR products from three individual mice for each genotype or muscle type and calculated skipping rates. We judged samples as being different from wild-type if a t-test indicated that the sample was unlikely to be from the wild-type distribution with P<0.05.

### Quantitative real-time PCR (qPCR)

qPCR was performed with SYBR Green PCR Master Mix (Invitrogen) and a BioRad CFX96 machine using 10 ng of cDNA in 20 µl total volume per reaction with the primers describe in Table S6. *Gapdh* was used for normalization and relative amounts of mRNA were calculated using the comparative CT (threshold cycle) method.

### Plasmids constructs, infection, and transfection

All constructs were fully sequenced before use. For RNA interference, shRNAs against *Rbfox1* (sh#1 CCCAGACACAACCTTCTGAAA; sh#2 CCGACAAATGTTTGGTCAATT) and the control non-silencing shRNA (TCTCGCTTGGGCGAGAGTAAG) in pLKO.1 were purchased from Open Biosystems (Huntsville, AL). Viruses were packaged in 293T cells and used to infect WT C2C12 cells according to manufacturer's instructions. Infected cells were selected with relative antibiotics (puromycin 0.5 µg/ml final) and maintained as a polyclonal population. Generation of C2C12-*EV* and C2C12-*FRG1* was described elsewhere [24]. For the experiments reported in Figure 10, *FRG1* ORF was cloned into the lentiviral vector pCCL.sin.cPPT.SV40ployA.eGFP.minCMV.hPGK.deltaNGFR.WPRE (EV1) [81] and *Rbfox1* ORF was cloned into the pBABE-puro retroviral vector (EV2). EV1 and FRG1 viruses were packaged in 293T cells using psPAX2 packaging plasmid and pMD2G envelope plasmid. The medium containing the lentiviral particles was harvested after 27 h, 34 h and 48 h, filtered by using a 0.22 mm filter unit and right after used to transduce WT C2C12 cells. After 48 h, transduced cells were 70 to 80% GFP positive. Then, EV2 and Rbfox1 viruses were packaged in 293T cells using VSV-G expression vector and gag/pol expression vector. The medium containing the retroviral particles was harvested after 27 h, 34 h and 48 h, filtered by using a 0.22 mm filter unit and right after used to transduce FRG1 cells. After 48 h, FRG1-EV2 and FRG1-Rbfox1 transduced cells were put under puromycin selection (puromycin 0.5 µg/ml final) for 5 days. The construct-containing mouse A713 *Rbfox1* ORF fused to a myc-epitope [82] was a kind gift of Dr. S. Kawamoto and was subcloned in pIRES-Puro (Clontech). *Calpain 3* lacking exon 6 ORF was obtained by RT-PCR from *FRG1* mice muscles and was cloned in pIRES-Neo (Clontech). To generate stable cells, 10 µg of *Fox1*-IRES-Puro, *Capn3 E6*-IRES-Neo plasmids and the appropriate empty vector controls were used to transfect C2C12 cells with Lipofectamine LTX (Invitrogen) following manufacturer's instructions. 48 hours post-transfection, cells were selected with puromycin or neomycin (neomycin 1 µg/ml final). Resistant cells were maintained as polyclonal populations.

### Protein extracts, immunoblotting, and antibodies

C2C12 and primary human muscle cells were lysed in SDS sample buffer at 95°C for 10 min. Muscle extracts were homogenized using buffer containing 0.125 M Tris-HCl pH6.4; 10% glycerol, 4% SDS, 4 M urea, 10% mercaptoethanol and 0.01% bromophenol blue (final pH of the buffer 6.8) according to the datasheet of CAPN-12A2 Novocastra antibody. Protein extracts were separated using a SDS-PAGE gel and transferred onto a nitrocellulose membrane (GE Healthcare). Immunoblots were incubated with primary antibodies against Rbfox1 (generous gifts from Dr. Douglas Black [83] and Dr. Thomas Cooper [84]), Calpain 3 (mouse monoclonal, 1∶250 #NCL-CAPN-12A2, Novocastra), HA (mouse monoclonal, 1∶500 #MMS-101, Covance) and anti-α tubulin antibody (mouse monoclonal, 1∶10,000, #049K4767, Sigma) as loading control. Subsequently, 1∶10,000 dilution of peroxidase-conjugated donkey anti-mouse IgG (#715-035-015. Jackson ImmunoResearch) was added. Detection was performed by super signal west pico chemioluminescence substrate (Thermo Scientific).

### Chromatin immunoprecipitation (ChIP) and RNA immunoprecipation (RIP)

Antibodies against FRG1 were kindly provided by DR. PL Jones [52]. Anti-RNA pol II (Millipore #17-620), anti-Myc monoclonal antibody (MMS-164P, Covance) or control mouse or rabbit IgG (715-035-015 and 011-000-003, Jackson ImmunoResearch) were also used. ChIP was performed as described in [21]. RIP displayed in Figure S6a was performed as [52]. RIP displayed in Figure 7d was performed as UV-RIP described in [21]. RIP displayed in Figure S6b, Figure 8 and Figure S7e was performed as ChIP described in [21]. The primers used are described in Table S6.

## Supporting Information

Figure S1 CD45 staining in *FRG1* mice. (a) Left panel: representative images of *vastus lateralis* staining against pan-hematopoietic marker CD45. Right panel: representative images of *vastus lateralis* Gömöri trichrome staining using a ×20 objective analysis. (b) Quantitative analysis of CD45 positive cells.
(TIF)

Figure S2 RT-PCR validation of splicing-sensitive microarrays. RT-PCR analysis was performed on RNA extracted from three independent *wild type* and *FRG1* mice at 4 weeks of age. For each gene, representative images as well as quantification of triplicate mice data with standard deviations are shown. For the quantification, RT-PCR products from three individual *FRG1* and control *WT* mice were quantified using the Typhoon and the skipping rates were calculated. Samples were judged as being different from *WT* if a t-test indicated that the sample was unlikely to be from the *WT* distribution with P<0.05. Numbers below images are the percentage of exon inclusion. Black boxes illustrate constitutive exons, white boxes alternatively spliced exons and double lines represent the affected intron.
(TIF)

Figure S3 Alternative splicing changes correlate with disease severity in different muscles. RT–PCR analysis of alternative splicing using RNA extracted from *vastus lateralis* (severely affected) and *biceps brachii* (mildly affected) muscles from *wild type* and *FRG1* mice at 4 weeks of age. Numbers below images are the percentage of exon inclusion. Black boxes illustrate constitutive exons, white boxes alternatively spliced exons and double lines represent the affected intron.
(TIF)

Figure S4 Alternative splicing changes in *FRG1* mice are not secondary to muscular dystrophy. RT-PCR analysis of alternative splicing using RNA extracted from *vastus lateralis* of gender, age and background-matched *WT*, *FRG1* and *mdx* mice. Numbers below images are the percentage of exon inclusion. Black boxes illustrate constitutive exons, white boxes alternatively spliced exons and double lines represent the affected intron.
(TIF)

Figure S5 Alternative splicing changes are a primary consequence of *FRG1* over-expression in tissue culture. (a) Specific *FRG1* over-expression was confirmed by real-time RT-PCR and immunoblotting using RNAs and proteins isolated from C2C12 muscle cells expressing a Flag-HA empty vector (*EV*) or a Flag-HA-tagged *FRG1* (*FRG1*) either in proliferating or differentiating C2C12 muscle cells. (b) RT-PCR analysis of alternative splicing using the same samples as in (a). Numbers below images are the percentage of exon inclusion. Black boxes illustrate constitutive exons, white boxes alternatively spliced exons and double lines represent the affected intron.
(TIF)

Figure S6 Altered transcripts are not direct FRG1 targets. (a) RIP experiments on control and over-expressing FRG1 C2C12 muscle cells using anti-FRG1 (HS2) or control IgG antibodies. (b) RIP experiments on control and over-expressing FRG1 C2C12 muscle cells using anti-FRG1 (HS2), anti-FRG1 (L-07 sc-101050, SCBT) or control IgG antibodies. Anti-FRG1 immunoprecipitated material did not show any enrichment versus control IgG. RT-minus control experiments showed the absence of DNA contamination.
(TIF)

Figure S7 *Rbfox1* knockdown causes alternative splicing changes similar to *FRG1* over-expression, *Rbfox1* over-expression causes opposite results. (a) Left: schematic representation of the regions analyzed showing the location of the putative Fox binding site (FBS) and RT-PCR analysis of alternative splicing in C2C12 muscle cells expressing *Rbfox1* shRNA#1 for genes with FBS. Right: RT-PCR analysis of alternative splicing in C2C12 muscle cells expressing *Rbfox1* shRNA#1 for genes without FBS. (b) Specific *Rbfox1* knockdown using a second shRNA was confirmed by real-time RT-PCR and immunoblotting using RNAs and proteins isolated from C2C12 muscle cells expressing a control shRNA or an shRNA specific for *Rbfox1* (shRNA#2). *Rbfox1* knockdown with shRNA#2 display a reduced myogenic differentiation. (c) RT-PCR analysis of alternative splicing in C2C12 muscle cells expressing *Rbfox1* shRNA#2 (#2) for genes containing putative FBS (left) or for genes without FBS (right). (d) RT-PCR analysis of alternative splicing in C2C12 muscle cells over-expressing *Rbfox1* (F1) for genes with putative FBS. Numbers below images are the percentage of exon inclusion. Black boxes illustrate constitutive exons, white boxes alternatively spliced exons and double lines represent the affected intron. (e) Selective *in vivo* association of Rbfox1 to target regions displaying putative Fox binding sites (FBS).
(TIF)

Figure S8 Real-time PCR analysis in proliferating (MB) or differentiating (MT) C2C12 muscle cells confirming selective *Capn3 E6-* isoform over-expression. Real-time RT-PCR analysis was performed on RNA extracted from proliferating (MB) or differentiating (MT) C2C12 expressing the empty vector or *Capn3 E6-* using primers specific for the *Capn3* isoform containing exon 6 (Capn3 cost) or the alternative splicing isoform lacking exon 6 (Capn3 E6-).
(TIF)

Table S1 Genes investigated at transcriptional levels.
(XLS)

Table S2 Genes investigated at splicing levels.
(XLS)

Table S3 Distribution of alternative splicing events and gene expression changes. (a) Top: Gene expression microarray data were filtered for *q* = 0 and fold change >1.5. Bottom: Summary of the genes altered only in *vastus lateralis*, in *vastus lateralis* and *biceps brachii* or only in *biceps brachii* at 4 or 13 weeks of age. Summary of the genes altered only at 4 weeks, at 4 and 13 weeks or only at 13 weeks of age in *vastus lateralis* or in *biceps brachii*. (b) Top: Splicing microarray data were filtered for *q* = 0 and |Sepscore|≥0.3. Significant splicing events were categorized by RNA processing mode. Bottom: Summary of the total events altered only in *vastus lateralis*, in *vastus lateralis* and *biceps brachii* or only in *biceps brachii* at 4 or 13 weeks of age. Summary of the total events altered only at 4 weeks, at 4 and 13 weeks or only at 13 weeks of age in *vastus lateralis* or in *biceps brachii*.
(XLS)

Table S4 Lists of transcripts identified by splicing-sensitive microarray that were validated by RT-PCR. Gene symbol and gene name is listed.
(PDF)

Table S5 List of primers used for the RT-PCR validation of the splicing-sensitive microarrays.
(TIF)

Table S6 List of primers used for real-time RT-PCR, RNA-IP, and ChIP.
(TIF)
