# Supplementary figures and images for: Rbfox1 Downregulation and Altered Calpain 3 Splicing by FRG1 in a Mouse Model of Facioscapulohumeral Muscular Dystrophy (FSHD)
Source: PLoS Genet. 2013 Jan 3;9(1):e1003186. doi: 10.1371/journal.pgen.1003186 (PMC3536703; doi:10.1371/journal.pgen.1003186)

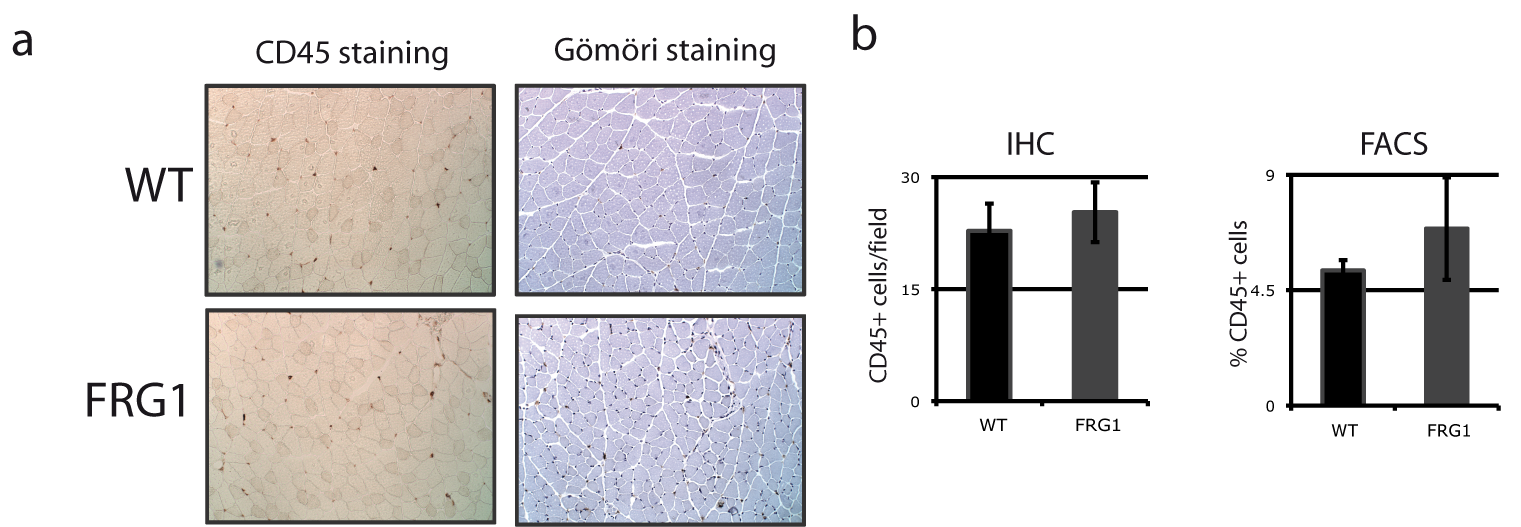

Supplement: Figure S1 — CD45 staining in FRG1 mice. (a) Left panel: representative images of vastus lateralis staining against pan-hematopoietic marker CD45. Right panel: representative images of vastus lateralis Gömöri trichrome staining using a ×20 objective analysis. (b) Quantitative analysis of CD45 positive cells. (TIF) [file pgen.1003186.s001.tif]

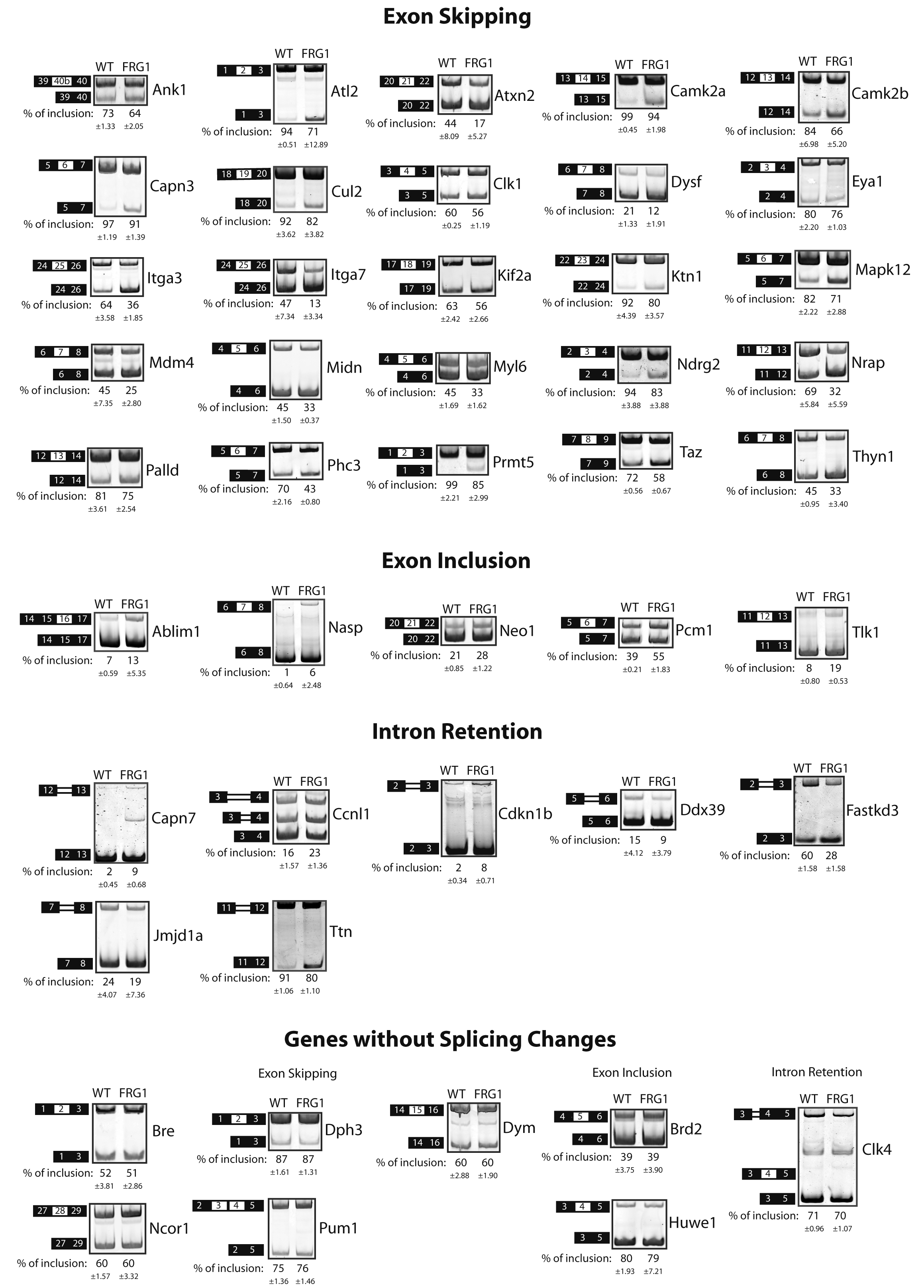

Supplement: Figure S2 — RT-PCR validation of splicing-sensitive microarrays. RT-PCR analysis was performed on RNA extracted from three independent wild type and FRG1 mice at 4 weeks of age. For each gene, representative images as well as quantification of triplicate mice data with standard deviations are shown. For the quantification, RT-PCR products from three individual FRG1 and control WT mice were quantified using the Typhoon and the skipping rates were calculated. Samples were judged as being different from WT if a t-test indicated that the sample was unlikely to be from the WT distribution with P<0.05. Numbers below images are the percentage of exon inclusion. Black boxes illustrate constitutive exons, white boxes alternatively spliced exons and double lines represent the affected intron. (TIF) [file pgen.1003186.s002.tif]

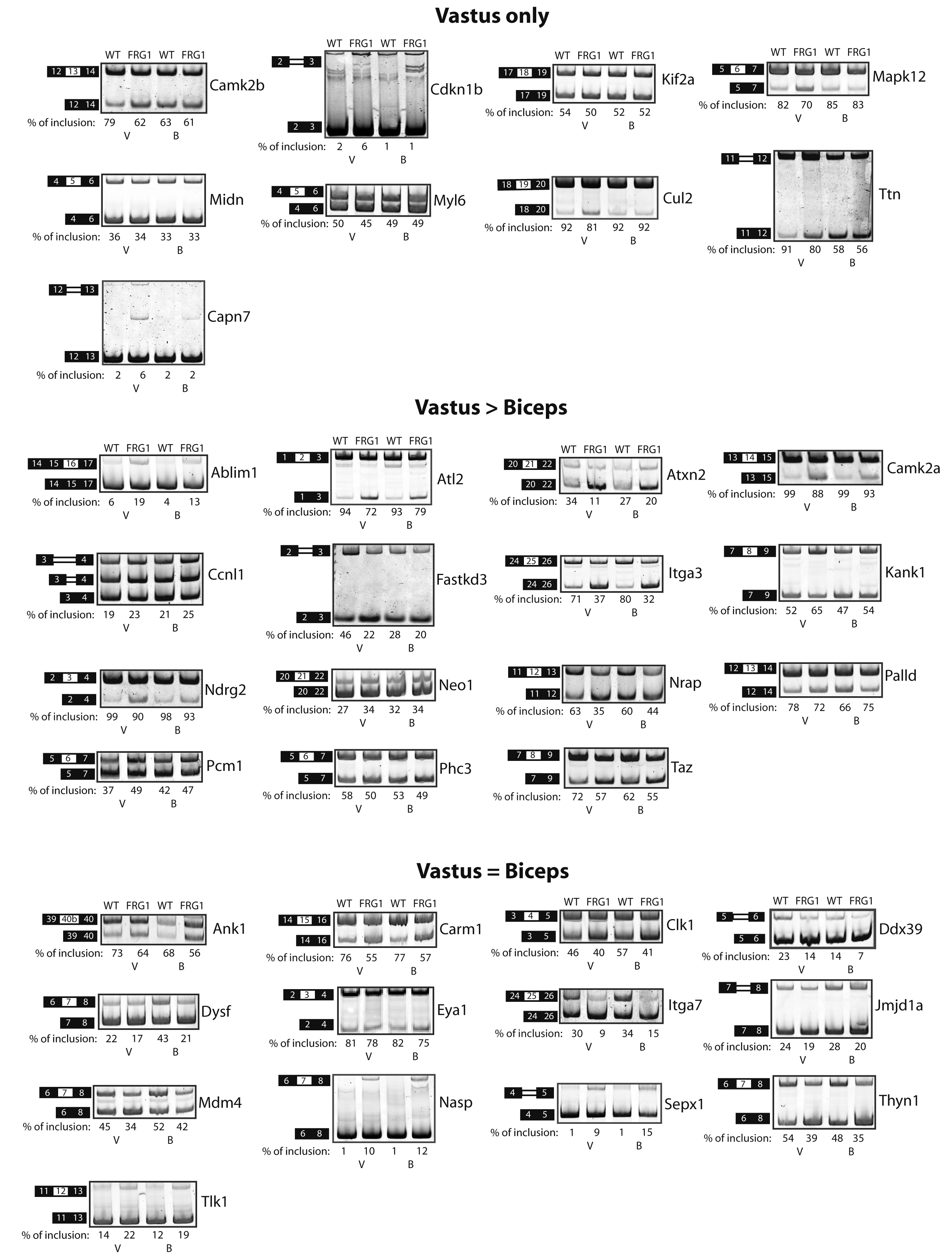

Supplement: Figure S3 — Alternative splicing changes correlate with disease severity in different muscles. RT–PCR analysis of alternative splicing using RNA extracted from vastus lateralis (severely affected) and biceps brachii (mildly affected) muscles from wild type and FRG1 mice at 4 weeks of age. Numbers below images are the percentage of exon inclusion. Black boxes illustrate constitutive exons, white boxes alternatively spliced exons and double lines represent the affected intron. (TIF) [file pgen.1003186.s003.tif]

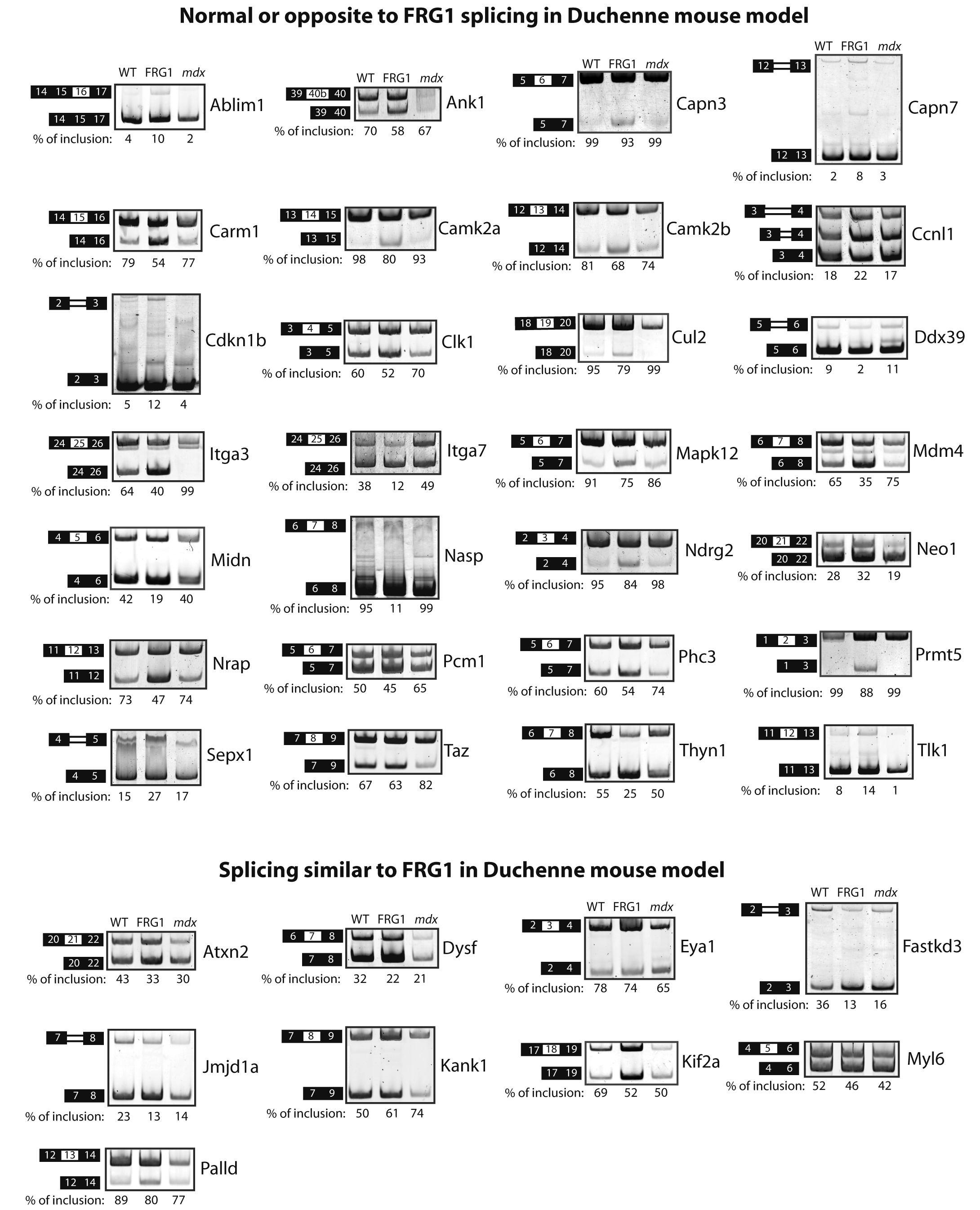

Supplement: Figure S4 — Alternative splicing changes in FRG1 mice are not secondary to muscular dystrophy. RT-PCR analysis of alternative splicing using RNA extracted from vastus lateralis of gender, age and background-matched WT, FRG1 and mdx mice. Numbers below images are the percentage of exon inclusion. Black boxes illustrate constitutive exons, white boxes alternatively spliced exons and double lines represent the affected intron. (TIF) [file pgen.1003186.s004.tif]

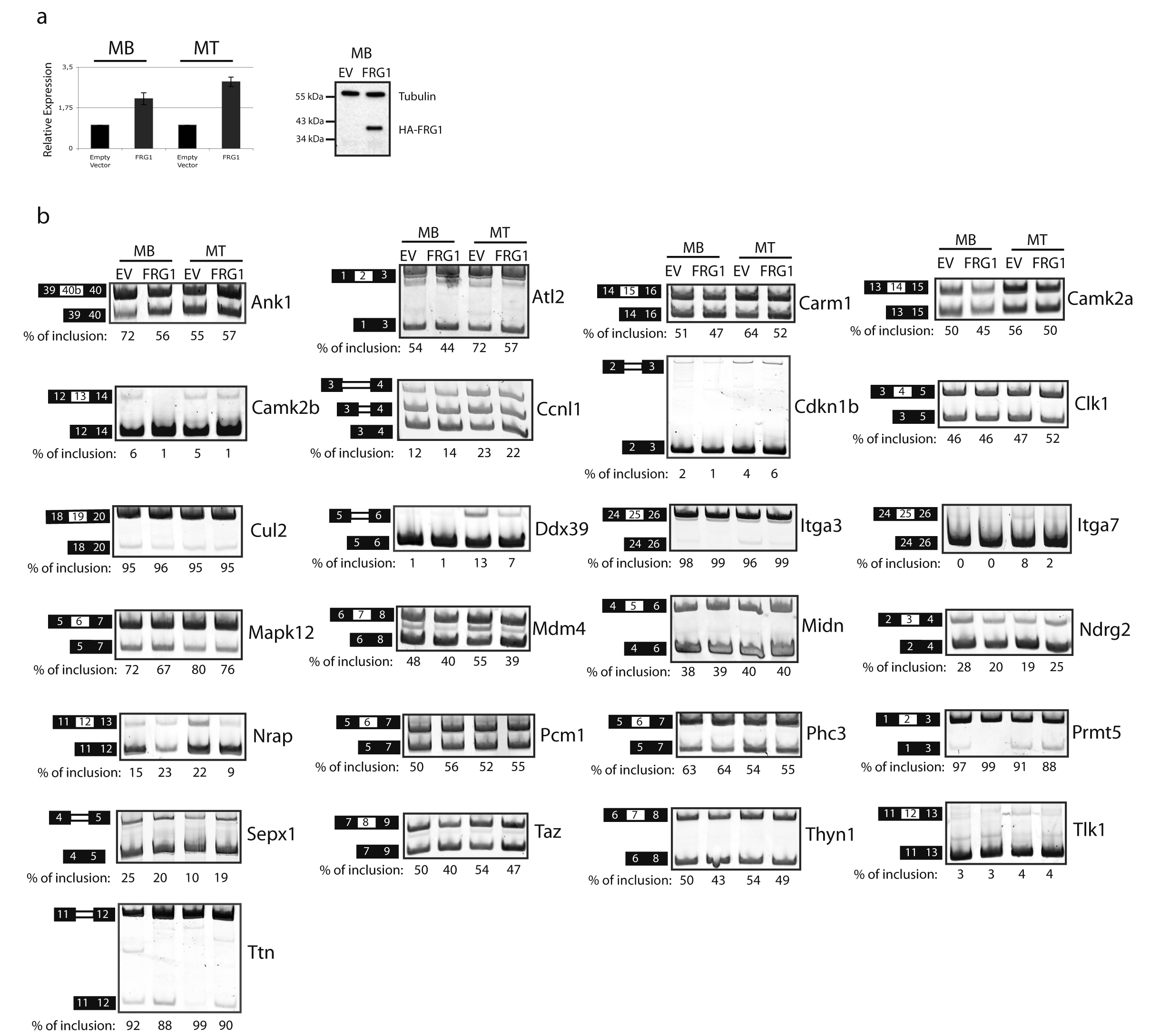

Supplement: Figure S5 — Alternative splicing changes are a primary consequence of FRG1 over-expression in tissue culture. (a) Specific FRG1 over-expression was confirmed by real-time RT-PCR and immunoblotting using RNAs and proteins isolated from C2C12 muscle cells expressing a Flag-HA empty vector (EV) or a Flag-HA-tagged FRG1 (FRG1) either in proliferating or differentiating C2C12 muscle cells. (b) RT-PCR analysis of alternative splicing using the same samples as in (a). Numbers below images are the percentage of exon inclusion. Black boxes illustrate constitutive exons, white boxes alternatively spliced exons and double lines represent the affected intron. (TIF) [file pgen.1003186.s005.tif]

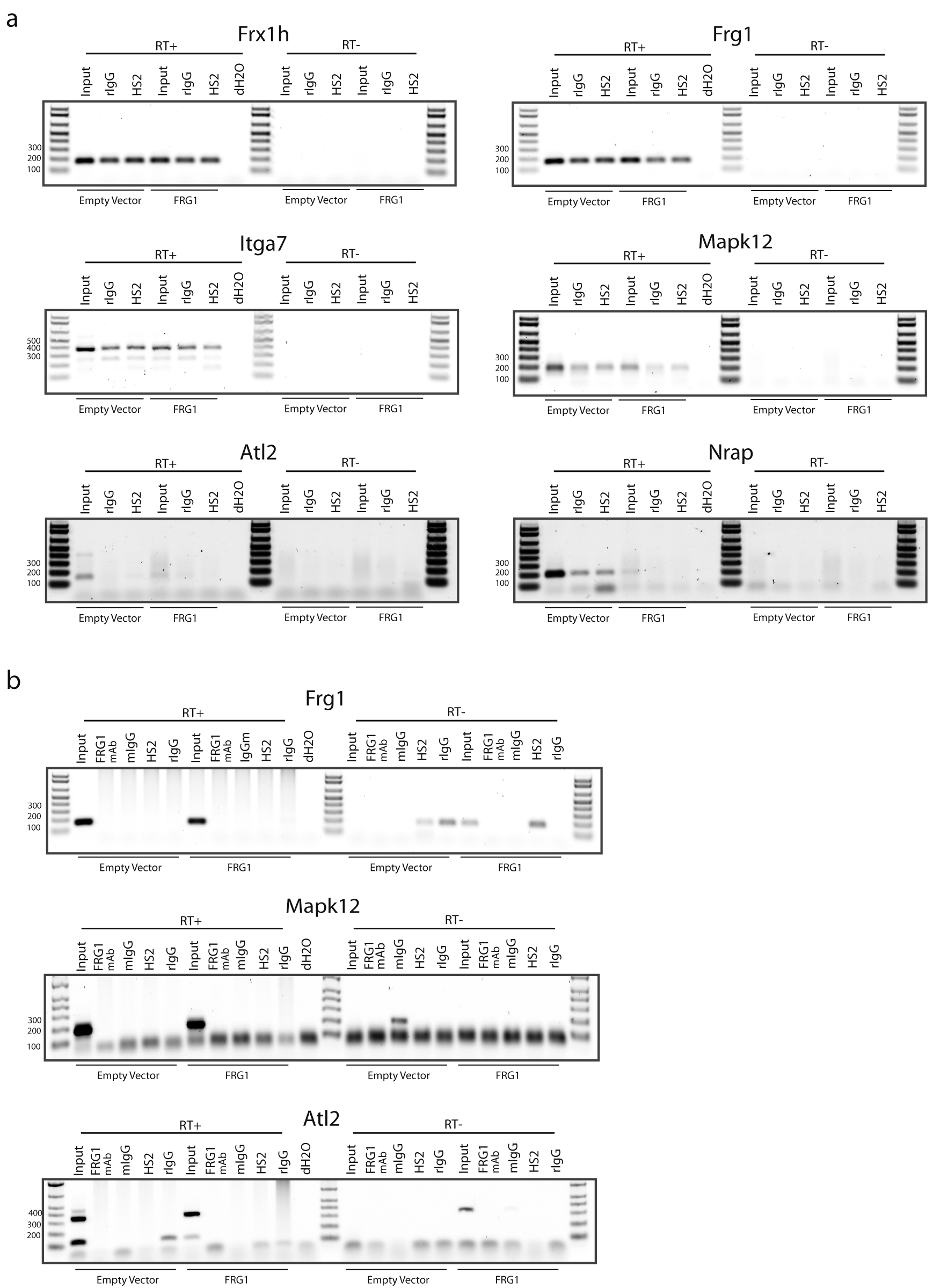

Supplement: Figure S6 — Altered transcripts are not direct FRG1 targets. (a) RIP experiments on control and over-expressing FRG1 C2C12 muscle cells using anti-FRG1 (HS2) or control IgG antibodies. (b) RIP experiments on control and over-expressing FRG1 C2C12 muscle cells using anti-FRG1 (HS2), anti-FRG1 (L-07 sc-101050, SCBT) or control IgG antibodies. Anti-FRG1 immunoprecipitated material did not show any enrichment versus control IgG. RT-minus control experiments showed the absence of DNA contamination. (TIF) [file pgen.1003186.s006.tif]

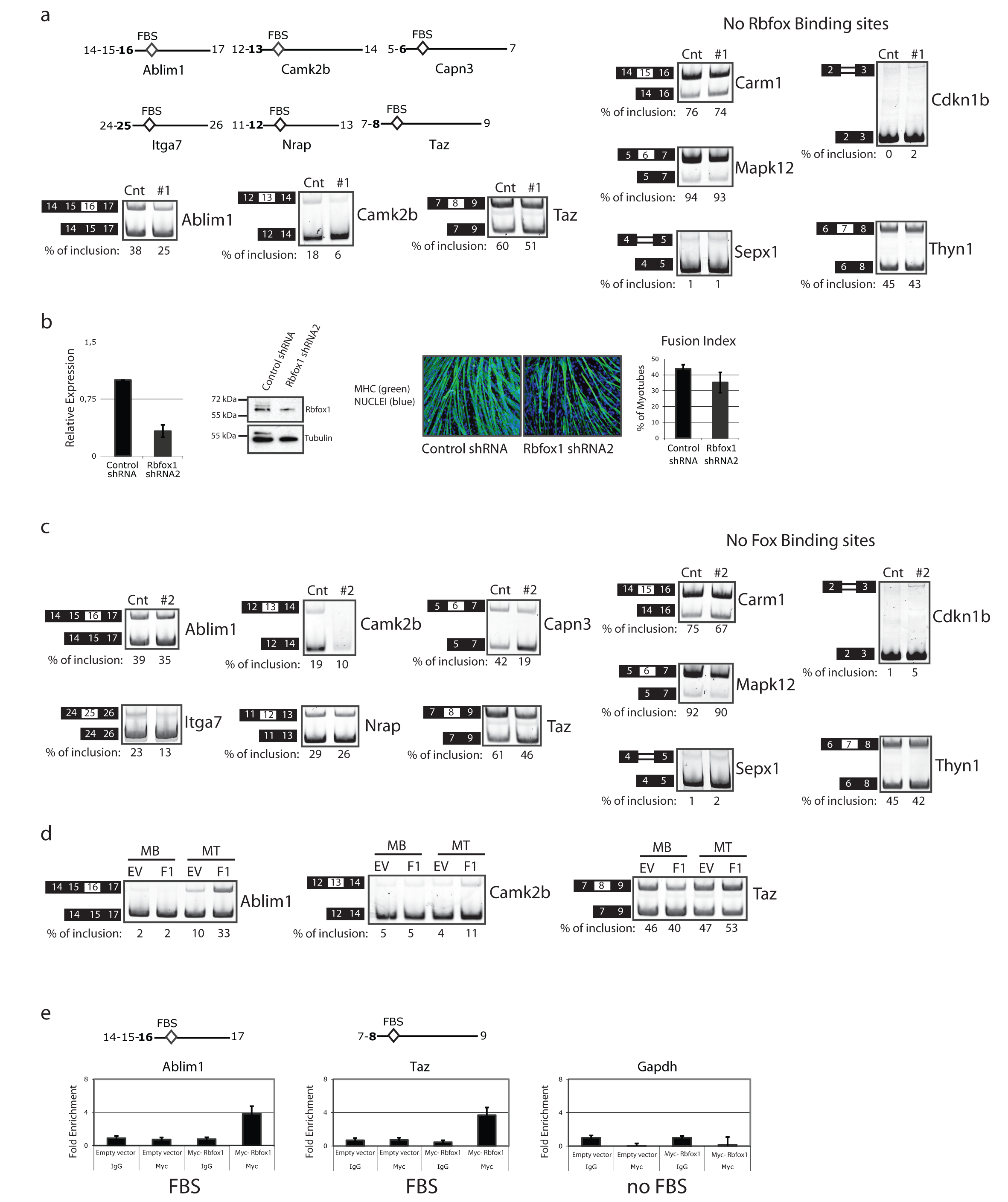

Supplement: Figure S7 — Rbfox1 knockdown causes alternative splicing changes similar to FRG1 over-expression, Rbfox1 over-expression causes opposite results. (a) Left: schematic representation of the regions analyzed showing the location of the putative Fox binding site (FBS) and RT-PCR analysis of alternative splicing in C2C12 muscle cells expressing Rbfox1 shRNA#1 for genes with FBS. Right: RT-PCR analysis of alternative splicing in C2C12 muscle cells expressing Rbfox1 shRNA#1 for genes without FBS. (b) Specific Rbfox1 knockdown using a second shRNA was confirmed by real-time RT-PCR and immunoblotting using RNAs and proteins isolated from C2C12 muscle cells expressing a control shRNA or an shRNA specific for Rbfox1 (shRNA#2). Rbfox1 knockdown with shRNA#2 display a reduced myogenic differentiation. (c) RT-PCR analysis of alternative splicing in C2C12 muscle cells expressing Rbfox1 shRNA#2 (#2) for genes containing putative FBS (left) or for genes without FBS (right). (d) RT-PCR analysis of alternative splicing in C2C12 muscle cells over-expressing Rbfox1 (F1) for genes with putative FBS. Numbers below images are the percentage of exon inclusion. Black boxes illustrate constitutive exons, white boxes alternatively spliced exons and double lines represent the affected intron. (e) Selective in vivo association of Rbfox1 to target regions displaying putative Fox binding sites (FBS). (TIF) [file pgen.1003186.s007.tif]

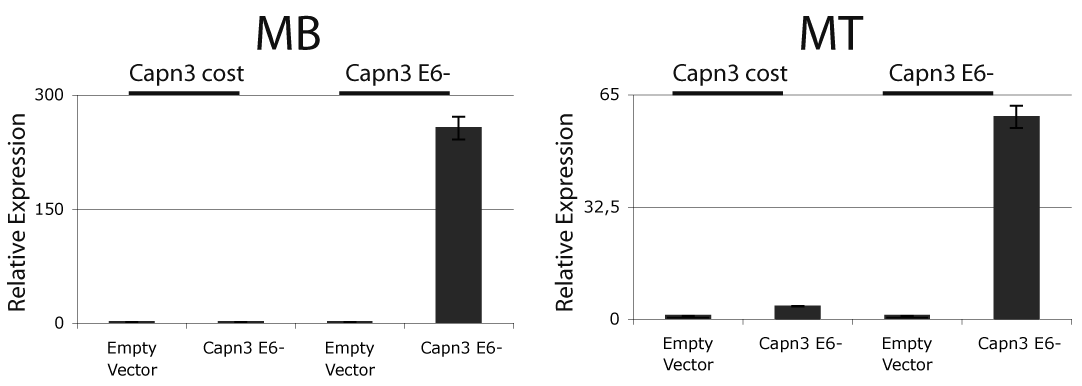

Supplement: Figure S8 — Real-time PCR analysis in proliferating (MB) or differentiating (MT) C2C12 muscle cells confirming selective Capn3 E6- isoform over-expression. Real-time RT-PCR analysis was performed on RNA extracted from proliferating (MB) or differentiating (MT) C2C12 expressing the empty vector or Capn3 E6- using primers specific for the Capn3 isoform containing exon 6 (Capn3 cost) or the alternative splicing isoform lacking exon 6 (Capn3 E6-). (TIF) [file pgen.1003186.s008.tif]

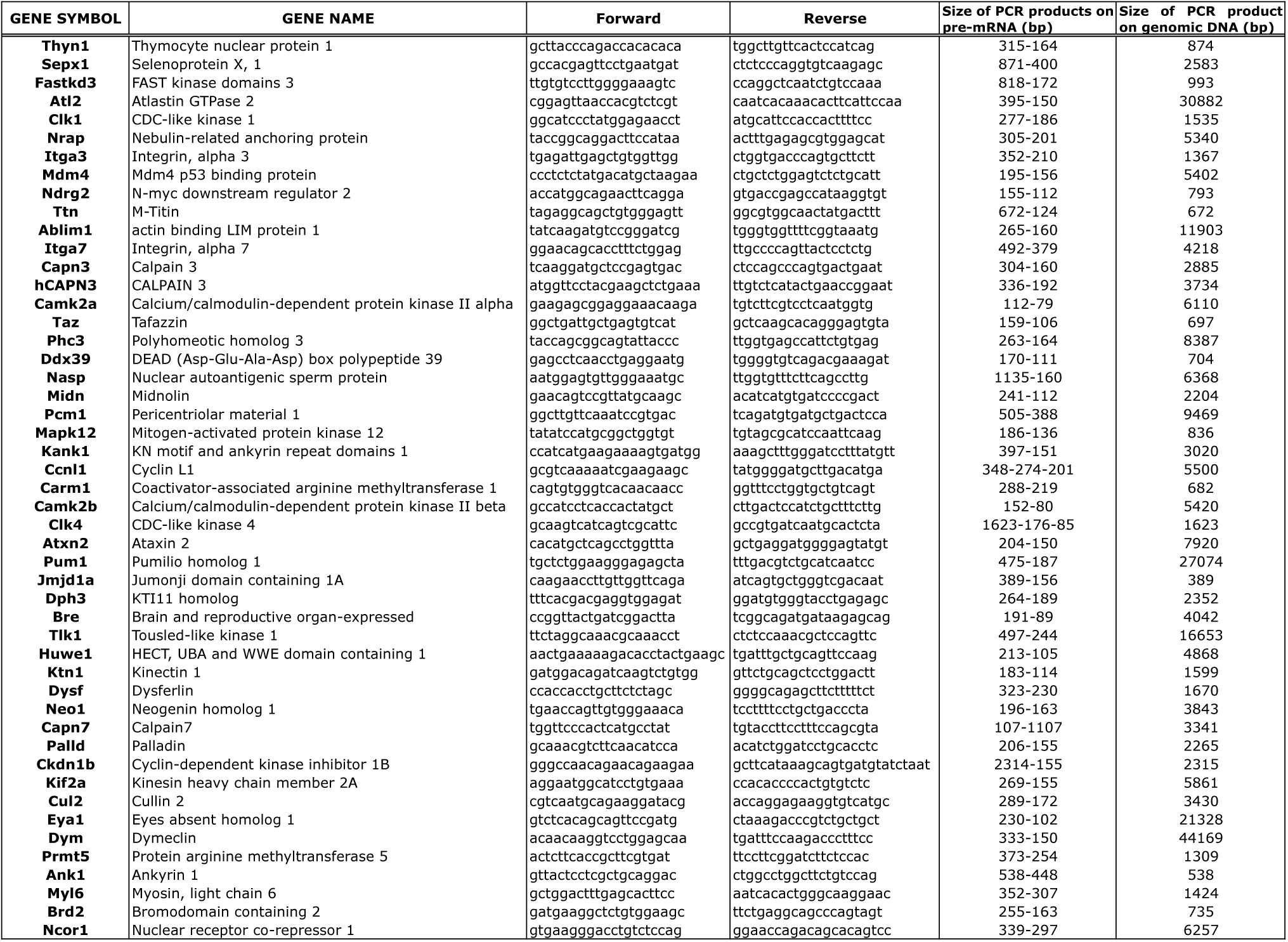

Supplement: Table S5 — List of primers used for the RT-PCR validation of the splicing-sensitive microarrays. (TIF) [file pgen.1003186.s013.tif]

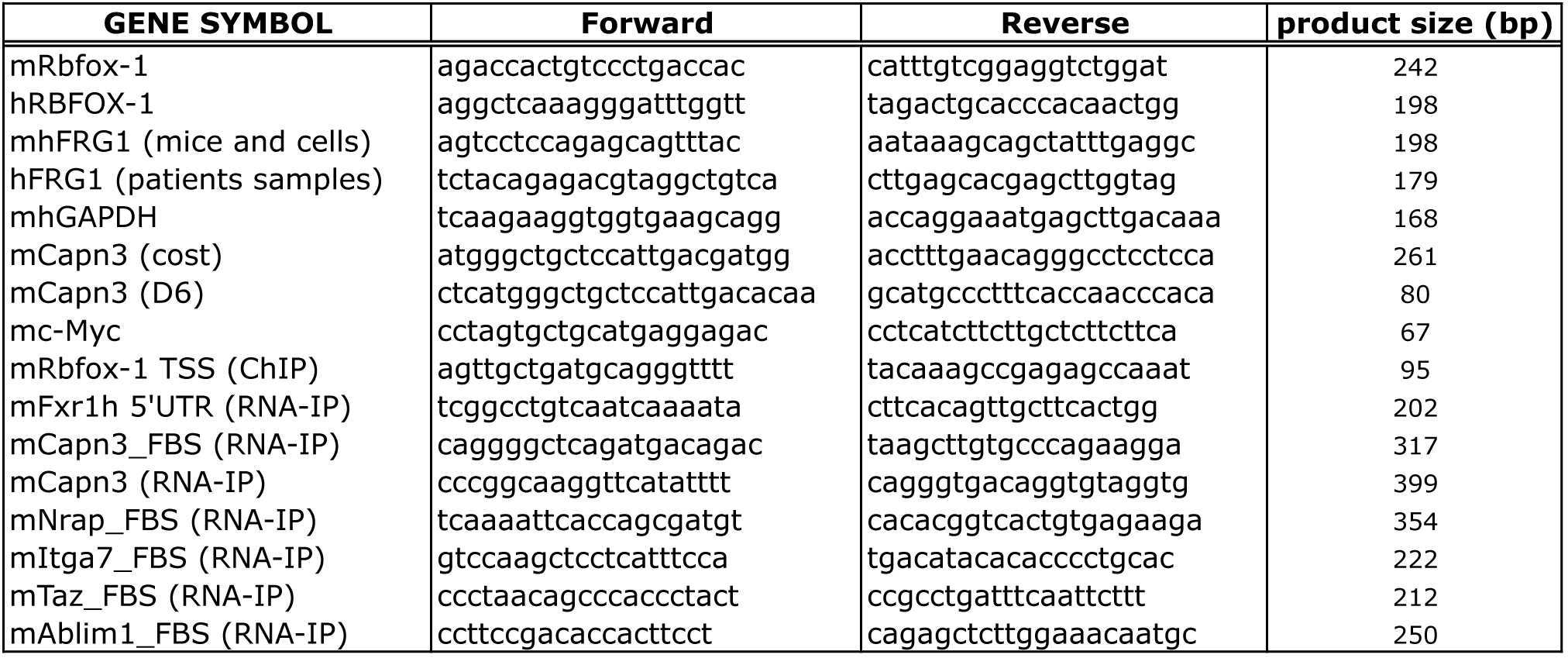

Supplement: Table S6 — List of primers used for real-time RT-PCR, RNA-IP, and ChIP. (TIF) [file pgen.1003186.s014.tif]
