# Supplementary material for: Rbfox1 Downregulation and Altered Calpain 3 Splicing by FRG1 in a Mouse Model of Facioscapulohumeral Muscular Dystrophy (FSHD)
Source: PLoS Genet. 2013 Jan 3;9(1):e1003186. doi: 10.1371/journal.pgen.1003186 (PMC3536703; doi:10.1371/journal.pgen.1003186)
Supplement: Table S4 — Lists of transcripts identified by splicing-sensitive microarray that were validated by RT-PCR. Gene symbol and gene name is listed. (PDF) [file pgen.1003186.s012.pdf]

GENE SYMBOL	GENE NAME
**Thyn1**	Thymocyte nuclear protein 1
**Sepx1**	Selenoprotein X, 1
**Fastkd3**	FAST kinase domains 3
**Atl2**	Atlastin GTPase 2
**Clk1**	CDC-like kinase 1
**Nrap**	Nebulin-related anchoring protein
**Itga3**	Integrin, alpha 3
**Mdm4**	Mdm4 p53 binding protein
**Ndrg2**	N-myc downstream regulator 2
**Ttn**	M-Titin
**Ablim1**	actin binding LIM protein 1
**Itga7**	Integrin, alpha 7
**Capn3**	Calpain 3
**Camk2a**	Calcium/calmodulin-dependent protein kinase II alpha
**Taz**	Tafazzin
**Phc3**	Polyhomeotic homolog 3
**Ddx39**	DEAD (Asp-Glu-Ala-Asp) box polypeptide 39
**Nasp**	Nuclear autoantigenic sperm protein
**Midn**	Midnolin
**Pcm1**	Pericentriolar material 1
**Mapk12**	Mitogen-activated protein kinase 12
**Kank1**	KN motif and ankyrin repeat domains 1
**Ccnl1**	Cyclin L1
**Carm1**	Coactivator-associated arginine methyltransferase 1
**Camk2b**	Calcium/calmodulin-dependent protein kinase II beta
**Clk4**	CDC-like kinase 4
**Atxn2**	Ataxin 2
**Pum1**	Pumilio homolog 1
**Jmjd1a**	Jumonji domain containing 1A
**Dph3**	KTI11 homolog
**Bre**	Brain and reproductive organ-expressed
**Tlk1**	Tousled-like kinase 1
**Huwe1**	HECT, UBA and WWE domain containing 1
**Ktn1**	Kinectin 1
**Dysf**	Dysferlin
**Neo1**	Neogenin homolog 1
**Capn7**	Calpain7
**Palld**	Palladin
**Ckdn1b**	Cyclin-dependent kinase inhibitor 1B
**Kif2a**	Kinesin heavy chain member 2A
**Cul2**	Cullin 2
**Eya1**	Eyes absent homolog 1
**Dym**	Dymeclin
**Prmt5**	Protein arginine methyltransferase 5
**Ank1**	Ankyrin 1
**Myl6**	Myosin, light chain 6
**Brd2**	Bromodomain containing 2
**Ncor1**	Nuclear receptor co-repressor 1
